# Rare earth elements distribution in topsoil from Ditrău Alkaline Massif area, eastern Carpathians, Romania

**DOI:** 10.1016/j.heliyon.2023.e13976

**Published:** 2023-02-24

**Authors:** Adriana Ion, Ana Cosac

**Affiliations:** Geological Institute of Romania, Radiometry Laboratory, 1 Caransebes st., RO-012271, Bucharest, Romania

**Keywords:** Topsoil, Rare earth elements, Spatial distribution, pH, Alkaline rocks

## Abstract

This paper gives an overview of the REEs distribution in topsoil from Ditrău Alkaline Massif area under influence of basic natural factors (parent material and soil acidity). Seventy-six soil samples were collected in accord with the most representative bedrock types and concentrations of the elements were determined using inductively coupled plasma mass spectrometry. The ΣREEs contents in soil developed on alkaline rocks ranges from 52.59 to 579.20 μg/g, with an average value of 235.76 μg/g, significantly higher than the average value of Earth's crust (179.7 μg/g). Y content varies between 5.50 and 58.80 μg/g with an average of 21.67 μg/g. The soils are enriched in LREEs (La to Eu) and depleted in HREEs (Gd to Lu) and Y. This trend is expressed by the wide variations of the LREEs/HREEs, (La/Yb)_ch_, (La/Sm)_ch_ and (Gd/Yb)_ch_ ratios. The REE chondrite - normalized plots show for most soils strongly negative anomalies for cerium and europium and positive anomalies for gadolinium and dysprosium. The pH of soils is generally acidic to weakly acidic and has an insignificant role in REEs fractionations in soil. The spatial distribution of REEs is strongly related to the lithology of the study area, displaying minor to negligible effects of enrichment factors and a low geoaccumulation index, corresponding to the lack of anthropic contamination. The distribution of the elements in topsoil tends to mimic elemental accumulation in the parental bedrock, with a potential to highlight mineralized zones.

## 1 Introduction

The rare earth elements (REEs) represent a geochemically coherent group with similar chemical and physical properties [1]. The rare earth elements (REEs) group is subdivided into light rare elements (LREEs) that include La, Ce, Pr, Nd, *Pm* (unstable, not occurring as a natural element), Sm, Eu and heavy rare earth elements (HREEs) comprising Gd, Tb, Dy, Ho, Er, Tm, Yb and Lu (Henderson et al., 1984). Yttrium is included in this group, as it has similar chemical properties with REEs and a geochemical behavior between dysprosium (Dy) and holmium (Ho) in most environments in the Earth's crust [2] Typical for all REEs is an oxidation degree of 3+, though europium has a stable divalent state and cerium a stable tetravalent state, strongly depending on the redox potential [3]. The actual behavior of REEs results from the interaction between their chemical environment and differences in their chemical properties determined by electronic ground state configurations and the so called “lanthanide contraction” – the regular decrease in ionic radius of these elements with the increase in atomic number [4]. The REEs are valuable geochemical tracers, for instance in the description of the chemical evolution of the earth's crust and the origins of sediments [[5], [6], [7], [8], [9]]. Geochemistry of REEs is often used as an indicator of weathering and soil forming processes [10].

Studies on rare earth elements in soils shows that the most of REEs originate from locally derived geological parent materials. The concentration of REEs in soils is in general small, varying considerably with bedrock types and source area [[11], [12], [13], [14]]. Elevated REEs values are generally indicative of soils derived from felsic rocks, especially intrusive ones [10,15]. In igneous environments, REEs are generally incompatible (partition into the melt over minerals) and are typically concentrated in accessory phases. Hence, these elements are enriched in melts that result either from very low degrees of partial melting or from extreme fractionation. In igneous rock series (diorite, gabrodiorite, granite, syenite, particularly nepheline syenite), the total content of REEs varies between 8 and 1977 mg/kg and decreases as the degree of basicity increases [16].

Several studies have reported that besides the parental materials, the concentrations of REEs in soils depends on additional factors, such as: the stability of primary REE-bearing minerals to weathering, the presence of a clay phase and the organic matter content, because during weathering mobilized REEs can be incorporated in these phases, physical and chemical characteristics of the soils [[17], [18], [19], [20], [21], [22]]. Other favorizing factors are the presence in soil of Fe and Mn oxyhydroxides and chloride, sulfate, phosphate anions [23,24].

REEs are typically concentrated in alkaline rocks and the concentration of REEs in young soils can essentially be a mean of the concentration and abundances of REE in parental rocks [25]. Accordingly, the Alkaline Massif of Ditrău, due to its petrographic diversity (hornblendite, diorite, syenite, nepheline syenite, monzonite, monzodiorite, aplite, alkali granite) presents unique opportunities for geochemical exploration of REEs in soil.

In spite of the extensive knowledge related to the petrology and mineral resources of the area, no systematic study was undertaken so far to investigate the distribution of element concentration in the soil and its possible constraints.

The aims of the present study were: (i) to evaluate REEs level and distribution in topsoil samples collected from the Ditrău masiff area; (ii) investigate the relationship among elements distribution and parental rocks, soil types and acidity of soil (pH).

The study was the first to determine the concentration of REEs in the surface soils of the Ditrău massif, and to draw spatial distribution maps for each element in the REEs group. The data provide from this work may be used to assess the potential mineral resource in the study area and anthropogenic influences.

## 2 Geological and pedological setting

The Alkaline Massif of Ditrău is located in south western of the Giurgeu Mountains (46^0^48^’^ N, 25^0^30^’^E) eastern Romania and, covers some 225 Km^2^. The climate is transitional temperate continental and the mean of annual temperature is around 5.90 °C, with around +30 °C maximum in summertime and minimum up to −35 °C in wintertime. In winter temperature inversions occur frequently, a phenomenon that contributes to early autumn mist and frost and late spring frost. Frost occurs for 160–165 days per year. The average value of precipitation is approx. 600 mm. The annual number of rainy days amounting to 100–108.

**Fig. 1:**
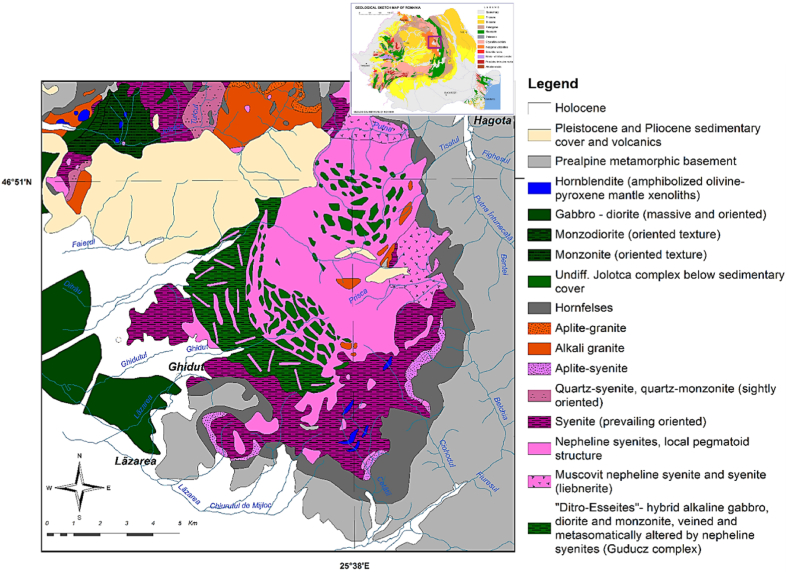
Geological map of Ditrău Alkaline Massif area (modified after [28]).

Geologically, the Alkaline Massif of Ditrău represents an intrusive alkaline body with an internal zonal structure, emplaced into pre-Alpine metamorphic basement rocks at the interior of the Eastern Carpathians (Fig. 1) [[26], [27], [28]]. The Ditrău massif has an elliptical shape, and is placed east of a geophysically located crustal fault (G8), the most important structural element in the study area. The Ditrău massif represent the border between the Crystalline-Mesozoic belt of the Eastern Carpathians to the East, and the Neogene volcanic belt to the West [29]. The age of the alkaline massif, according to precise U–Pb zircon and titanite dating for syenite and ^40^Ar/^39^Ar dating for amphibole and pyroxene-rich cumulate shows that the massif has crystallized between 238.6 ± 8.9 and 225.3 ± 2.7 Ma (Middle – Late Triasic) [30]. The data mentioned are in good agreement with other published values [31,32].

The intrusive complex has a distinct structure in which more basic rocks occur in the western part, whereas an arcuate zone extending north to south - east on the eastern part is dominated by nepheline syenites [33]. Part of the massif is covered by Neogene andesitic pyroclastics and lava flows and the Pliocene - Pleistocene sediments of the Gheorgheni and Jolotca Basins.

Petrographically, the Alkaline Massif of Ditrău is unique, characterized by a great diversity of magmatic rocks with gradual transitions amongst them. Thus, in this petrological heterogeneity, the center of the massif is formed by nepheline syenites surrounded by syenites and monzonites. The hornblendites, gabbro/hornblendites, alkali diorites, monzodiorites, monzosyenites and alkali granites occurs in north-eastern and north-western marginal area of the massif. Particularly, small discret ultramafic bodies occur in the Jolotca area and comprise kaersutite-bearing peridotites, piroxenites and hornblendites, as well as alkali gabbro [33]. Here, the diorites, hornblendites and hornblende gabbros are earlier intrusive phases incorporated in younger syenite and granite [31,33]. Throughout the whole complex the rocks are cut by randomly-oriented late-stage dykes exceeding 1 m in width, which include tinguaites, phonolites, nepheline syenites, microsyenites, aplites and lamprophyres [[34], [35], [36], [37], [38], [39], [40]]. In the east and south-eastern part of the massif alkali diorites and, occasionally, alkali gabbros associate with syenites, like in Jolotca area, are forming the Güdücz complex. For both similar magmatic suites, Streckeisen used the term of "Ditro-essexites” [41]. In the Ditrău area, the essexites occur in the central - south - western part of massif, as hybrid alkaline gabbros, alkali diorites, diorites and monzonites, veined and metasomatically altered by nepheline syenites [28]. In the marginal sectors of the complex, especially towards the southern, south-eastern and eastern contact transformed nepheline syenites and syenites (muscovitized and liebeneritized are largely developed), producing features relevant to this study [42,43].

The great number of different rocks which occur in the massif area and the complicated field relationships among these rocks imparts a high petrographic complexity to the area that is accompanied by a large diversity of mineral occurrences. Mineralogical studies carried out at Ditrău have shown that REEs are concentrated in a number of REE-bearing minerals, such as allanite, chevkinite-perrierite, cerite, monazite, fluorapatite, xenotime, bastnäsite, parisite, synchysite, hidroxilbastnäsite, thorite, thorogummite, pyrochlore, columbite, fergusonite, euxenite, aeschynite, zircon, fluorine, titanite, garnet. Most REEs bearing minerals are preferentially enriched in LREEs, and depleted in HREEs, such as monazite, bastnäsite, parisite, allanite, pyrochlore, columbite, aeschynite, apatite, thorite, while xenotime, zircon, garnet. Titanite and fluorine preferentially host HREEs and yttrium [34,[42], [43], [44], [45], [46], [47], [48]]. Mostly of these minerals occur in veins and disseminated mineralization areas (Jolotca, Belcina, Hereb - Csanad, Pricske, Putna, Creanga Halasag). Furthermore, the rare earth elements are present in the major silicates as isomorphic substituents of major cations, such as: microcline, albite, oligoclase, augite, cancrinite, hornblende, epidote.

**Fig. 2:**
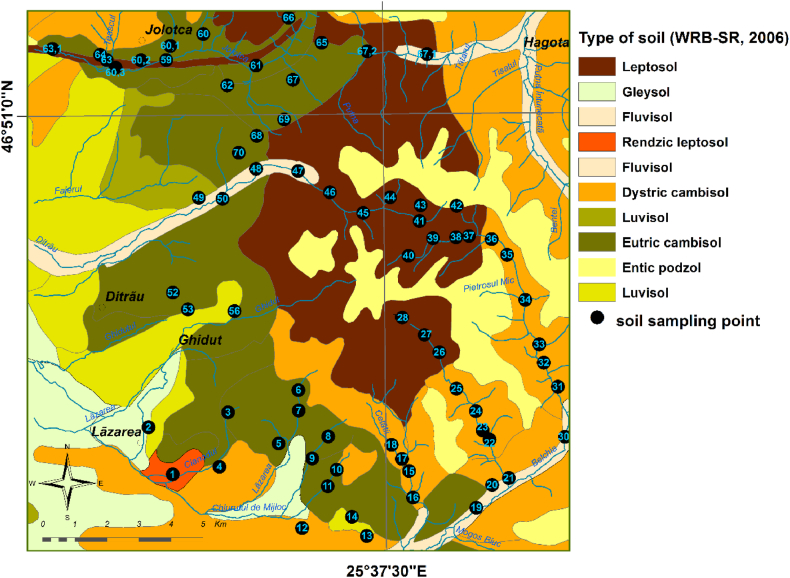
The soil map of Ditrău Alkaline Massif with the sampling location.

The main types of soil genetically associated with these rocks, according to the Romanian Soil Taxonomic Classification [49,50] and WRB-SR-2006 (World Reference Base for Soil Resources) [51]. Are: leptosols, eutri cambisols, dystri cambisols, prepodzols, rendzic leptosol, aluviosols, preluvosols, luvosols and gleysols (Fig. 2).

The leptosol, eutric cambisol, dystric cambisol and entic podzol covers most surface of the massif, being developed on alkaline rocks and metamorphic rocks that surround the Ditrău massif. Rendzic leptosols occupy a small area in southwestern part of massif near the Lăzarea village and were formed on crystalline limestones (marble) and dolomites. All these soils have undeveloped soil profile and are, often, thin and rich in skeletal material represented by unaltered rock fragments [52].

Luvisols occurs in the western edge of the study area on Pleistocene and Pliocene sedimentary formations, and Holocene formations from the Mureș Basin. They are moderately or strongly differentiated textural soils, which causes unfavorable aero-hydric characteristics on the profile (the clay content decreases with depth) [52].

Fluvisols are developed on small areas in the meadows of the Ditrău, Jolotca, Putna Întunecoasă and Belcina brooks, whereas gleysols are present in Mureș valley meadow and in submontane depressions surrounding Lăzarea and Chiruț villages. These soils are formed under conditions of prolonged excess moisture. The parent materials are represented by fluvial deposits with a clayey texture [52].

## 3 Materials and methods

### 3.1 Sampling

Soil samples in the Ditrău Alkaline Masiff and adjacent area, which includes the contact zone with the country-rocks (hornfelses) and the metamorphic rocks that immediately surround the massif, were collected in accordance to the European field manual for agricultural and grazing land soil [53]. A number of 70 soil samples were collected from the investigated area (263 Km^2^) in relation with the most representative bedrock types (Fig. 2). Soil sampling was done by manual borehole drilling at a depth range of 0–15 cm. At each point, five samples were collected from the corners and center of a square measuring 10 m on each side. The permanent grass cover was excluded. After collecting mentioned are were mixed, resulting one composite sample, weighing about 1.5 kg, which was placed into a plastic bag.

### 3.2 Sample preparation and analysis

During laboratory preparation, soil samples were dried at room temperature and then sieved by Teflon sieves with a 2 mm, respectively 0.63 mm mesh [54]. The fraction of 0.63 mm was quartered and homogenized in a mill to a grain size below 0.15 mm.

For determination of REEs, subsamples reduced to 0.1 g were digested in PTFE Teflon beakers using an acid mixture of HNO_3_ - HF – HClO_4_, at ∼200 °C for about 1 h. For monitoring instrumental deviation during a measuring cycle an internal standard of Rh solution (1 ml of μg ml^−1^) was added. After digestion, samples were evaporated to dryness on a hot plate. Then the residue was dissolved in 10 ml HCl to obtain the sample solution. The REEs concentration in soil samples from digested solution was determined by inductively couple plasma - mass spectrometry ICP-MS (X Series Thermo Elemental). The detection limit of ICP-MS is between 0.01 and 0.004 μg/l.

The pH of soil samples was determined in a CaCl_2_ solution: 16 g soil (2 mm fraction) was mixed with 40 ml of al dilute 0.01 M solution of CaCl_2_ (for dispersion), shaken for 60 min and immediately followed by the reading of pH. The pH values were measured using a pH-meter (WTW - 7310 series), after calibration with two buffer solution (pH = 7 and pH = 4) [55].

### 3.3 Exploratory data analysis

Statistic parameters of geochemical data set were calculated using the SPSS 17 software. Each variable (concentration of element) was estimated in terms of minimum, maximum, standard deviation (SD), variance (V), skewness (A), kurtosis (E), median absolute deviation (MDA), geometric mean (GM), median (Md), and coefficient of variation (CV). The correlation analysis was performed for REEs concentrations and pH values to determine relationships between these parameters based on the Pearson's coefficients. The assumptions of normality for the geochemical data set were evaluated by Kolmogorov – Smirnov test (p).

### 3.4 Geochemical background

The geochemical background was calculated with the equation suggested by Ref. [56].

*Geochemical background = Median ± 2 MAD*; where MAD is median absolute deviation.

### 3.5 Enrichment factors (EF) and geoacumulation index (Igeo)

In order to assess the origins of REEs (geogenic or anthropogenic) accumulated in topsoils, the results are analyzing using the Enrichment factor (EF) and Geoaccumulation index (Igeo).

The Enrichment Factor [57] compares the relative concentration of element accumulated in topsoils to that in crust (concentration of Post-Archean Australian Shale (PAAS) [58]. The Enrichment Factor (EF) was determined using formula [59]:

(1) ([REE]sample/[REE]PAAS)/([Y]sample/[Y]PAAS)

where REE and Y concentration in the soil sample and Post-Archean Australian Shale (PAAS) are considered [58]. Yttrium was adopted as a suitable reference element due to its low mobility in all environmental conditions [57]. Six classes of contamination are generally distinguished on the basis of the Enrichment Factor: non-enrichment (EF < 1), low enrichment (1≤EF < 2), moderate enrichment (2≤EF < 5), significant enrichment (5≤EF < 20), very high enrichment (20≤EF < 40), and extremely high enrichment (EF ≥ 40) [60].

The Geoaccumulation index (Igeo) it is complementary to the Enrichment factor and indicates, numerically, the level of topsoils pollution. This index (I_geo_) was calculated using the equation proposed by Ref. [61].

(2) Igeo = ln Cn/1.5*Bn

where: Cn is the concentration of the element in the soil; Bn is the geochemical background value; 1.5 is a correction factor owing to possible variation in background values due to lithological effects [62]. Müller [63] has defined seven classes of Geoaccumulation Index, in the following order: Class 0 (Igeo≤0) – uncontaminated soil; Class 1 (0<Igeo<1) – uncontaminated to moderately contaminated soil; Class 2 (1<Igeo<2) – moderately contaminated soil; class 3 (2<Igeo<3) – moderately to heavily contaminated soil; Class 4 (3<Igeo<4) – heavily contaminated soil; Class 5 (4<Igeo<5) – heavily to extremely contaminated soil and Class 6 (Igeo>5) –extremely contaminated soil.

### 3.6 REE – normalized patterns and Ce, Eu, Gd, Tb and Ho anomalies

REE concetrations were normalized by chondrites values [64]. This normalization was used to compare the REEs concentrations estimated to different parent materials [65]. The (La/Yb)_ch_, (La/Sm)_ch_, (Gd/Yb)_ch_, and LREEs/HREEs rations reflects the fractionation among LREEs and HREEs, where ch means concentrations normalized to chondrites [64]. The cerium, europium, gadolinium, terbium and holmium anomalies were calculated using the relations: δCe = 2Ce/(La_N_ + Pr_N_), δEu = 2Eu/(Sm_N_ + Gd_N_), δGd = 2Gd/(Eu_N_ + Tb_N_), δTb = 2Tb/(Gd_N_ + Dy_N_), δHo = 2Ho/(Dy_N_ + Er_N_) [66,67].

### 3.7 Geochemical maps

Spatial distribution maps for the analyzed chemical elements were generated in ArcGIS 10.02 software, using the inverse-distance weighting (IDW) interpolation method.

## 4 Results and discussion

### 4.1 Statistical analysis

Table 1 presents the descriptive statistics of REEs and pH in topsoil samples. The statistic parameters (standard deviation (SD), variance (V), skewness (A); Kurtosis (E); median absolute deviation (MAD); geometric mean (GM), median (Md), Kolmogorov - Smirnov normality test (K–S), and coefficient of variation (CV) show different variations of the REEs contents in the topsoil layers of Ditrău Alkaline Massif. The geometric mean (GM) is the average value or mean that represents the geochemical data set's central tendency, and it was used to depict the average concentration of REEs and Y. The soil content the LREEs (La, Ce, Pr, Nd, Sm, Eu) and HREEs (Gd, Tb, Dy, Er, Ho, Yb, Lu) are characterize by enriched of cerium (18.10–235.70 μg/g), lanthanum (14.49–181.10 μg/g), neodymium (9.30–106.8 μg/g), relative enriched of praseodymium (3.50–39.0 μg/g), and relative depleted in HREEs. For all REEs. The geometric mean values (GM) are closer to the median value (Md), suggesting an asymmetric data distribution pattern (Table 1). The standard deviation (SD) summarizes the variability in the geochemical dataset, and denotes that several values of elemental concentrations are further away from the mean, more precisely, it marks the frequency of extreme values. The standard deviation (SD) values are higher for Ce (42.68), La (37.14), Nd (19.37), and relative higher for Pr (6.84), Y (11.13) indicating an asymmetric distribution of these elements in the investigated soils. The skewness parameter (A) suggests a positive asymmetry to the right for all variables, more pronounced for Eu (1.69), Pr (1.57), Sm (1.43) and Er (1.54), which are closer to the kurtosis (E) positive values. Kolmogorov - Smirnov normality test (K–S) shows a lognormal distribution for most of the elements analyzed (p < 0.05). Only Dy, Tb and Y have a normal distribution in topsoils. The variance (V) expresses a very high degree of dispersion for Ce (1821.9), La (1381.42), Nd (375.21), and moderate for Pr (46.83). The coefficient of variation (CV) expresses in relative terms the variability of the same property for similar values of variance and different means. High values of the coefficient of variation (CV) suggest a spatially inhomogeneous distribution of REEs, while low values of the CV reflect a spatially homogeneous distribution [68]. The spatial variability was high to moderate for all REEs (CV values between 44.28 and 62.51%). The median absolute deviation (MDA) together with the median (Md) values compose the principal parameters used in calculating the geochemical background of REEs in the investigated soils, as well as the threshold values for each element of the rare earth elements group (Table 2).

**Table 1:** Descriptive statistics for REEs, contents (in μg/g) and pH in topsoils.

Element	N	Min	Max	Mean	SD	V	A	E	MDA	GM	Md	K–S	CV
La	70	14.49	181.10	66.11	37.17	1381.42	1.28	1.37	29.02	57.40	53.45	0.001	56.22
Ce	70	18.10	235.70	87.98	42.68	1821.90	1.34	2.58	30.17	79.33	86.05	0.000	48.52
Pr	70	3.50	39.00	12.86	6.84	46.83	1.57	3.35	5.05	11.80	11.85	0.000	53.23
Nd	70	9.30	106.80	39.01	19.37	375.21	1.24	2.36	14.52	34.14	36.15	0.049	49.65
Sm	70	1.70	22.60	7.76	3.97	15.76	1.43	3.48	2.82	6.98	7.50	0.002	51.16
Eu	70	0.50	6.00	1.78	1.11	1.24	1.69	3.57	0.80	1.54	1.50	0.000	62.51
Gd	70	1.00	15.80	6.11	3.05	9.29	1.13	1.44	2.25	5.28	5.65	0.015	49.9
Tb	70	0.20	2.60	0.98	0.49	0.24	0.93	1.31	0.54	0.87	1.00	0.031	50.19
Dy	70	1.40	15.50	5.74	2.91	8.48	1.16	1.98	2.21	5.05	5.35	0.200*	50.76
Ho	70	0.30	2.80	0.96	0.53	0.28	1.07	1.35	0.41	0.83	0.90	0.003	54.82
Er	70	0.70	7.00	2.77	1.37	1.87	0.88	0.83	1.09	2.44	2.55	0.200*	49.41
Tm	70	0.10	1.50	0.46	0.23	0.05	1.54	4.82	0.17	0.40	0.40	0.001	50.73
Yb	70	0.50	7.30	2.86	1.27	1.60	0.85	1.27	0.98	2.58	2.70	0.015	44.24
Lu	70	0.10	0.90	0.35	0.16	0.03	0.94	1.32	0.69	0.32	0.30	0.000	44.48
Y	70	5.50	58.80	21.67	11.13	123.93	0.99	1.16	8.71	18.97	19.25	0.089	51.38
ΣREEs	70	52.59	579.20	235.76	111.10	12344.18	1.11	1.24	83.70	198.53	216.25	0.005	47.13
ΣLREEs	70	48.29	530.40	215.50	103.93	10800.66	1.14	1.22	78.09	194.55	203.00	0.004	48.23
ΣHREEs	70	4.30	53.20	20.23	9.53	90.80	1.07	1.80	7.33	18.12	18.10	0.088	47.1
pH	70	3.69	7.38	4.86	0.66	0.43	0.75	1.71		4.87	4.86	.200*	13.54

N - number of samples; Min – minimum; Max – maximum; SD – standard deviation; V – variance; A – skewness; E − Kurtosis; MAD – median absolute deviation; GM – geometric mean; Md – median; K–S - Kolmogorov - Smirnov normality test; CV – coefficient of variation.

**Table 2:** Geochemical background and threshold of REEs in topsoils from Ditrău area and reference soil.

	Study area			Reference soil^a^	
Element	Geochemical background	Geochemical threshold	Mean content	Geochemical background	Geochemical threshold
	μg/g	μg/g	μg/g	μg/g	μg/g
La	53.45 ± 58.04	111.49	66.11	44.3 ± 18	62.4
Ce	86.06 ± 60.34	145.44	87.98	55 ± 45	100
Pr	11.85 ± 10.1	21.9	12.86	8.5 ± 5.2	13.7
Nd	36.15 ± 29.04	65.19	39.01	58 ± 24.8	82.6
Sm	7.50 ± 5.64	13.14	7.76	5.1 ± 2.6	7.8
Eu	1.50 ± 1.6	3.10	1.78	1.1 ± 0.4	1.5
Gd	5.65 ± 4.5	10.15	6.11	3.5 ± 1.8	5.3
Tb	1.00 ± 1.08	2.08	0.98	0.5 ± 0.4	0.97
Dy	5.35 ± 4.42	9.77	5.74	4.3 ± 2.6	6.8
Ho	0.90 ± 0.82	1.72	0.96	0.3 ± 0.2	0.5
Er	2.55 ± 2.18	4.73	2.77	1.4 ± 0.6	2.0
Tm	0.4 ± 0.34	0.74	0.46	0.2 ± 0	0.2
Yb	2.70 ± 1.96	4.66	2.86	1.5 ± 0.8	2.2
Lu	0.30 ± 1.38	1.68	0.35	0.24 ± 0	0.24
Y	19.25 ± 17.42	36.65	21.67	17 ± 16.4	33.4

a Soil developed on alkaline rocks [73].

Higher values of statistical parameters (GM, SD, V, CV) for REEs are observed in samples of leptosols, dystric cambisols and eutric cambisols derived from nepheline syenite, syenite, hornblendite, gabbro – diorite and monzodiorite; probably generated by heterogeneity of the concentrations displayed in these soils.

### 4.2 Individual REEs

**Fig. 3:**
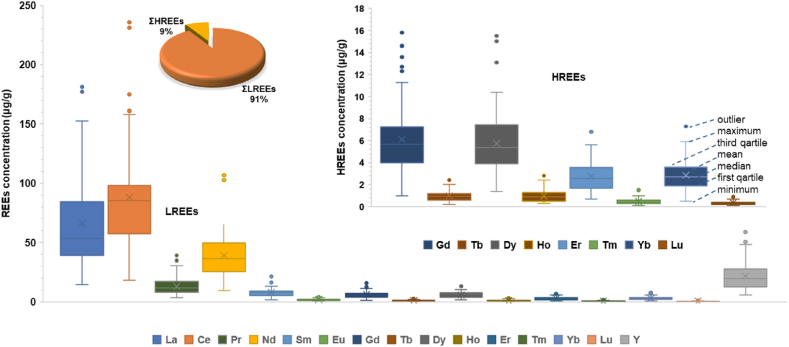
Box diagrams of REEs contents in topsoil samples.

The individual distribution of REEs in topsoils from Ditrău Alkaline Massif area are presented in Fig. 3.

According to the geochemical data (Fig. 3, Tables 1 and S1), the mean of REEs individual concentration in topsoils was observed to decrease in the following order: Ce > La > Nd > Pr > Sm > Gd > Dy > Yb > Er > Eu > Ho > Tb > Tm > Lu. The abundance of REEs in Ditrău topsoils complies with the Oddo-Harkin rule, by decreasing with increasing atomic number, while elements with odd atomic numbers are less abundant than their neighbors with even atomic number [10].

Ce, La and Nd are the most abundant REEs in topsoils, with 87.98 μg/g, 66.1 μg/g, respectively 39 μg/g mean contents. The individual content of REEs for the remaining REEs significantly decreases to mean values varying from 12.9 μg/g for Pr down to 0.7 μg/g for Lu. For yttrium, the mean content is 21.7 μg/g with a range from 5.5 to 58.8 μg/g.

The study area is considered unpolluted, the soils are forest soils, uncultivated, young, with undeveloped profile, thin and rich in skeletal material represented by unaltered rock fragments. Based on this field observations, is difficult to identify anthropogenic additions of REEs and, as a result the geochemical background of REEs in topsoils from Ditrău Massif reflecting natural processes; the degree of enrichment or depletion is completely dependent on the compositional and mineralogical features of the parent material. Consequently, the concentrations of REEs in the topsoils originated from alkaline rocks represent the natural background of these elements. The calculated values for the geochemical background and threshold values are shown in Table 2. Excepting terbium and lutetium, all REEs show concentration values exceeding the geochemical threshold in several samples.

For instance, in samples with IDs 27 and 28, Ce and La concentrations exceed the geochemical threshold. Here, cerium has the highest measured concentrations (231.1 μg/g and 235.7 μg/g) by far exceeding the threshold value (145 μg/g). Cerium and lanthanum enrichment in topsoils is generated by metasomatic syenites enriched in REE, U, Th, Zr [69,70] that occur in Prișica Peak area.

The geochemical threshold for all REEs (from La to Lu and Y) is overstepped in sampling points 60.2, 60.3, 63 and 63.1. The maximum concentrations of La, Pr, Nd and Sm were determined in topsoil sampling point no. 63. La has a concentration of 181.1 μg/g, Pr - 39.00 μg/g, Nd – 106.8 μg/g and Sm - 22.60 μg/g, values exceeding geochemical thresholds: La - 111.49 μg/g, Pr – 2.09 μg/g, Nd – 65.19 μg/g, and 13.14 for μg/g Sm. In sampling point no. 63.1 maximum concentrations for Eu (6.00 μg/g), Gd (15.80 μg/g), Dy (15.50 μg/g), Ho (2.80 μg/g), Er (7.00 μg/g), Tm (1.50 μg/g), Yb (7.30 μg/g) and Y (58.50 μg/g), were identified, as compared to the geochemical threshold values of Eu - 3.10 μg/g, Gd – 10.15 μg/g, Dy – 9.77 μg/g, Ho - 1.72 μg/g, Er - 4.72 μg/g, Tm - 0.78 μg/g, Yb (4.66 μg/g) and Y (36.65 μg/g). In this area (Jolotca), the REEs-enriched topsoils are genetically associated with REE + Nb + Mo vein mineralization hosted in ultramafic and mafic rocks [[42], [43], [44]].

Slight outrunning of threshold value for all REEs were recorded in all sampling points that cover the study area. This trend is typical for soils derived from alkaline rocks, REE-enrichment in soils is due to presence of REE-rich minerals resistant to weathering and occurrence of hydroxide complexes in such environments [15,71,72].

Table 2 compares geochemical background and threshold values obtained in the analyzed soils with geochemical data reported as reference natural background for soils originating from alkaline rocks [73] The REEs geochemical background for the Ditrău massif topsoils are visibly increased compared to the reference geochemical background, probably as an effect of REEs mineralization's hosted in the massif rocks.

In relation to the mean content, the means of individual REEs content in investigated topsoils are relatively high in comparison with their concentrations in similar environments (Table 3). The concentration of each element exceeds the values given for topsoils in Europe and Sweden [11,54]. The values obtained on Ditrău Massif are higher than the mean values reported by Ref. [74] for different soil types of various countries. Excepted lutetium, with averages comparable to those in the upper continental crust (UCC) cited by Rudnick and Gao (2003), all REEs from investigated soils have contents visibly higher than those reported for UCC, particularly for La, Ce, Nd, Pr and Sm. Average yttrium contents are also comparable to reference values [8,11,54].

**Table 3:** The REEs and Y concentration in investigated topsoil samples compared with similar environments.

Element mean (μg/g)	Study area	Topsoil, Europe [54]	Topsoil, Sweden [11]	Soil [74]	UCC [8]	
La	66.11	27.7	25.19	26.10	31	
Ce	87.98	52.2	37.67	48.70	63	
Pr	12.86	6.50	5.94	7.60	7.1	
Nd	39.01	22.4	15.14	19.50	27	
Sm	7.77	4.28	2.98	4.80	4.7	
Eu	1.78	0.85	0.65	1.23	1.0	
Gd	6.11	4.20	3.09	6.03	4.0	
Tb	0.98	0.64	0.47	0.71	0.7	
Dy	5.74	3.79	3.79	3.65	3.9	
Ho	0.96	0.72	0.61	1.08	0.83	
Er	2.77	2.10	1.88	1.58	2.3	
Tm	0.46	0.31	0.28	0.46	0.3	
Yb	2.86	2.09	2.01	2.06	2.0	
Lu	0.35	0.31	0.30	0.34	0.32	
Y	21.67	21	25	23^a^	21	

UCC - upper continental crust.

a Mean Y contents for uncultivated soil.

### 4.3 Individual REEs enrichment and geoaccumulation index (Igeo)

**Fig. 4:**
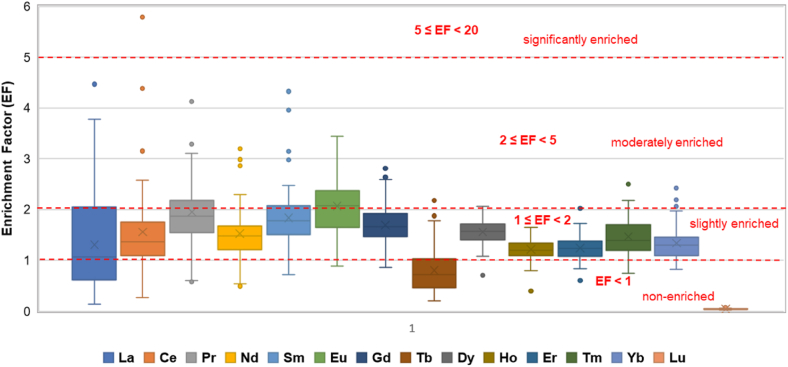
Box plot of EFs for REEs contents in topsoil samples.

**Fig. 5:**
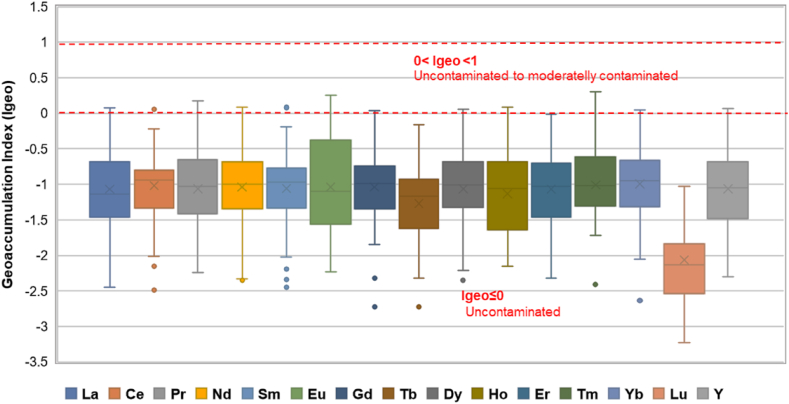
Box plot of Igeo for REEs contents in topsoil samples.

Natural variations in the abundance of REEs bearing minerals have an important effect on REEs distribution and accumulation in soils [10]. Therefore, the enrichment factor (EF) and Geoaccumulation index (Igeo) have been calculated to evaluate the enrichment degree of elements in soils and to assess the possible presence and intensity of anthropogenic contaminant on the surface soil. The enrichment factor (EF) and geoaccumulation index (Igeo) computed for all REEs studied in topsoil samples from Ditrău massif are shown in Fig. 4 and Fig. 5 and are listed in Tables 4, S2 and S3.

The mean of EF ranged from 2.06 for Eu to 0.04 for Lu, while maximal EF were for Ce (5,74), La (4.47), Pr (4.14), Nd (3.14), Sm (4.32), Eu (3.44), Gd (2.81), Tb (2.18), Dy (2.06), Ho (1,65), Er (2.02), Tm (2.54), Yb (2.42) and Lu (0.21). Most sampling points (Fig. 4) showed values of enrichment factors between 1 and 2 (1 ≤ EF < 2) in almost of all analyzed REEs. These values indicate a slightly enriched character. Terbium is the single element that has in the majority of sampling points a factor less than 1 (EF < 1) that does not indicate enrichment. According to data from Table 4 and Fig. 4 - moderate LREEs enrichments (2 ≤ EF < 5) were observed in the same topsoil samples developed from alkaline rocks. One single sampling point yielded an enrichment factor greater than 5 (EF = 5.74) indicating a significant enrichment in cerium. According to Ref. [75] EF values above 10 are considered indicators for anthropogenic signal of the nominated element, our data displaying enrichment factors close to 1 that signal a crustal parent.

**Table 4:** Calculated Enrichment Factor (EF) and Geoacummulation index (Igeo) for REEs in topsoil samples.

	EF													
	La	Ce	Pr	Nd	Sm	Eu	Gd	Tb	Dy	Ho	Er	Tm	Yb	Lu
Max.	4.47	5.79	4.14	3.19	4.32	3.44	2.81	2.18	2.06	1.65	2.02	2.54	2.42	0.12
Min.	0.13	0.26	0.57	0.49	0.72	0.88	0.86	0.20	0.70	0.40	0.60	0.74	0.82	0.02
Mean	1.30	1.56	1.95	1.53	1.83	2.06	1.69	0.80	1.56	1.21	1.24	1.47	1.34	0.04
	**I**_**geo**_													
	La	Ce	Pr	Nd	Sm	Eu	Gd	Tb	Dy	Ho	Er	Tm	Yb	Lu
Min.	−2.45	−2.49	−2.24	−2.35	−2.45	−2.23	−2.72	−2.72	−2.35	−2.15	−2.32	−2.41	−2.64	−3.23
Max.	0.08	0.08	0.17	0.09	0.14	0.25	0.04	−0.16	0.06	0.08	−0.01	0.30	0.04	−1.03
Mean	−1.07	−1.02	−1.06	−1.04	−1.06	−1.04	−1.04	−1.27	−1.06	−1.13	−1.07	−1.01	−1.00	−2.06

The geoacumulation index (Igeo) is a sensitive indicator for contamination of studied topsoils; the results (Fig. 5) for all REEs indicated that soil samples were uncontaminated (Igeo≤0). More than 90% of the Igeo values are negative, only in a few samples the values are slightly greater than 0 (0<Igeo<1) suggesting uncontaminated to moderately contaminated topsoils.

The quantification of the contamination parameters (EF and Igeo) indicates a natural accumulation of REEs in topsoils and denote that the REEs in the studied topsoils were mainly derived from the weathering processes of the parent rocks.

### 4.4 Sum of REEs

The mean of total REEs content (ΣREEs - from La to Lu) in the analyzed soils is high (235.76 μg/g), varying from 52.59 μg/g to 579.20 μg/g, *i. e.* higher than the average content reported in Earth's crust (179.7 μg/g) [76]. Considering the values obtained for contamination parameters (EF and Igeo), these results potentially represent the effect of two factors, namely: relatively high contents of REEs in parent materials and REEs enrichment during soil profile formation [10,[77], [78], [79]].

LREEs have the most substantial contribution in establishing the total content of REEs in soils; approximately 90% of the ΣREEs is given by LREEs (Fig. 3). The total content of light earth elements (ΣLREEs –La to Eu) has an average of 215.49 μg/g, recording an extensive variation from 48.19 μg/g to 530.4 μg/g. The mean content obtained for the sum of light rare earth elements often exceeds by far the average in the Earth's crust (159.2 μg/g) [76] It is agreed that the LREEs are less mobile than HREEs and this peculiarity causes LREEs enrichment and HREEs depletion as the intensity of weathering increases [10,80].

The total content of heavy rare earth elements (ΣHREEs – Gd to Lu) represent up to 10% of the total content of REEs, with an average of 20.23 μg/g in the range from 4.03 μg/g to 20.23 μg/g. The mean of ΣHREEs is comparable to their average in Earth's crust (20.5 μg/g) [76].

These data demonstrate that LREEs enrichment in all soil samples is a consequence of weathering process. This abundance pattern of REEs in soils matches that of the Ditrău Alkaline Massif; the rocks of massif being described as enriched in LREEs and depleted in HREEs [42]. In addition, typical for rocks which occur at Ditrău is the enrichment in cerium and depletion in lanthanum, a pattern matched by soils that developed on these rocks [42].

## 5 Discussion

### 5.1 REEs distribution in relationship with soil and rock types

**Fig. 6:**
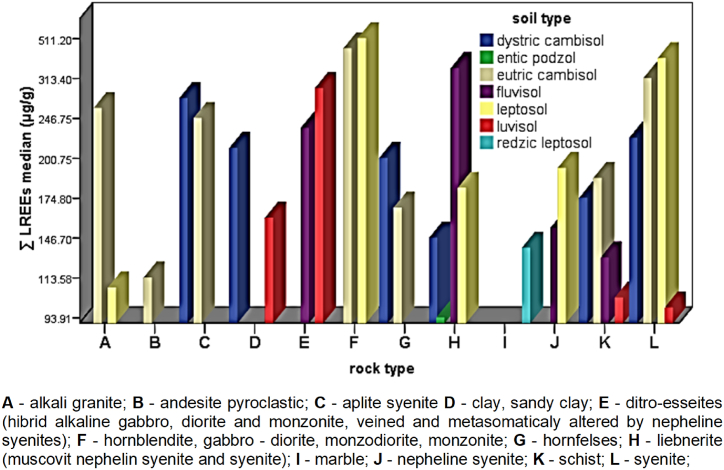
Distribution of ΣLEEs in soils according to the parent material.

**Fig. 7:**
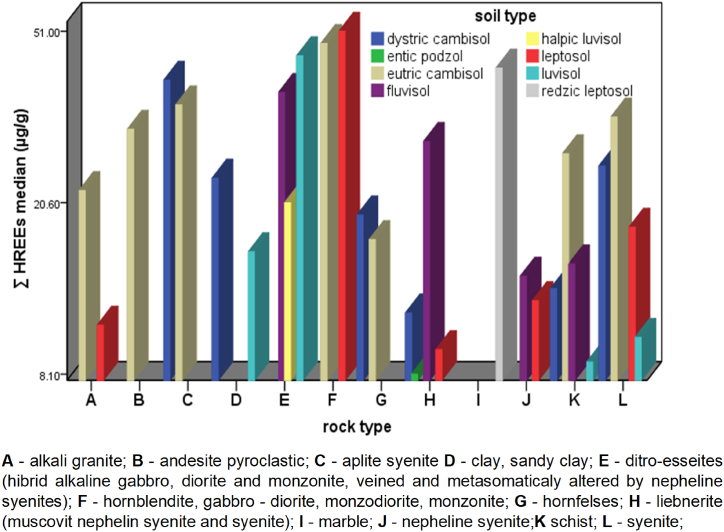
Distribution of ΣHREEs in soils according to the parent material.

The ΣREEs content in soil types of Ditrău Alkaline Massif decreases in the following order: Leptosols > Eutric cambisols > Fluvisols > Luvisols > Dystric cambisols > Redzic leptosoils > Entic podzols > Gleysols. The higher values content of REEs are observed in Leptosols and Eutric cambisols, 508.05 μg/g and 465 μg/g respectively. ΣLREEs follows the same order, whereas for ΣHREEs a different trend can be observed: Leptosols > Eutric cambisols > Luvisols > Dystric cambisols > Redzic leptosoils > Fluvisols > Entic podzols > Gleysols. The wide variations in REEs content indicate differences in the concentration of REEs in soils studied, the type of parent material being the main factor that governs these differences. The ΣLREEs and ΣHREEs according to the parent materials and soil types are shown in Fig. 6 and Fig. 7.

The distribution of ΣLREEs and ΣHREEs in relation to the type of the underlying rock vary significantly within and among different parent materials. In topsoils of the Ditrău Alkaline Massif, the ΣLREEs (Fig. 6) content decreases depending on the parent material in the following order: (hornblendite, gabbro-diorite, monzodiorite, monzonite) > syenite > liebenerite (muscovite nepheline syenite and syenite) > ditro–essexite (hybrid alkaline gabbro, diorite and monzonite, veined and metasomatically altered by nepheline syenites) > aplite syenite > alkali granite > nepheline syenite > schists > andesite pyroclastic > clay, sandy clay > marble. The ΣLRREs show higher average contents (530.4 μg/g) in soils derived from mafic, ultramafic rocks and syenites, nepheline syenites (488.9 μg/g). The ΣHRREs (Fig. 7) content broadly follows the order given by ΣLREEs, except for soils developed on marble, dolomite (31.10 μg/g) and andesite pyroclastics (25.80 μg/g). Here, the average content of ΣHREEs is comparable to that of soils developed on mafic rocks, respectively syenites.

The results obtained indicate that the parental material, especially alkaline rocks, is a determinant factor in the distribution of rare earth elements in soils, independent of soil types, physical – chemical characteristic and weathering degree; in according to similar results [77,[80], [81], [82]].

### 5.2 Spatial distribution of REEs

**Fig. 8:**
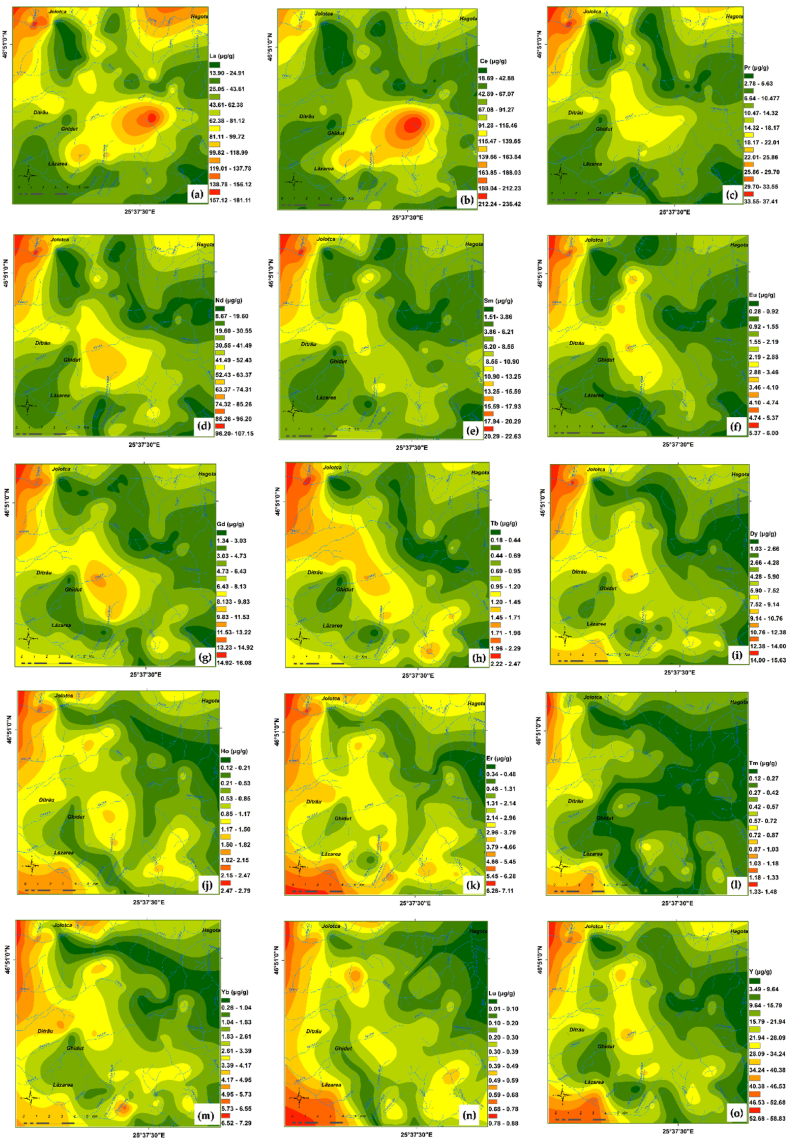
Individual spatial distribution of REEs (μg/g) in topsoil from investigated area: (a) La; (b) Ce; (c) Pr; (d) Nd; (e) Sm; (f) Eu; (g) Gd; (h) Tb; (i) Dy; (j) Ho; (k) Er; (l) Tm; (m) Yb; (n) Lu; (o) Y.

**Fig. 9:**
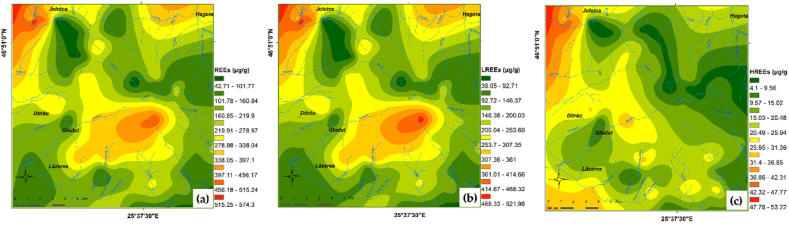
Spatial distribution of REEs (μg/g) in soils from investigated area: (a) REEs; (b) LREEs; (c) HREEs.

The spatial distribution map of REEs is useful in identifying high REEs concentrations areas and possible source of this elements. The maps for individual REEs are shown in Fig. 8 (a) to (o). The ΣREEs, ΣLREEs and ΣHREEs spatial distribution maps are reproduced in Fig. 9 - (a)–(c).

The REEs geochemical maps are a particularly useful tool in assessing possible sources of enrichments and identifying areas with increased concentrations of REEs in correlation with the geological substrate. Multielement geochemical maps (ΣREEs, ΣLREEs and ΣHREEs) (Fig. 9 contribute to the recognition of better expressed geochemical systems, while monoelemental maps (Fig. 8) ring details related to concentration peaks and offsets, practically indicating the sensitivity of the elements to the dispersion factors.

The spatial distribution analysis of REEs (Figs. 8 and 9) highlights the association of higher REEs concentrations with soil profiles developed on basic and ultrabasic rocks, as well as with those developed on metasomatic rocks. The lower contents were observed in soil derived from sedimentary rocks.

All LREEs display marked similarities in distribution and allow the contouring of some areas with higher LREEs contents, as follows:

i) a first area placed in the north-eastern parts of massif (Jolotca area) with the highest values for LREEs, especially cerium and lanthanum. Here, the geochemical halos have direct filiation with the better-developed REE + Nb + Mo vein mineralization hosted in ultramafic and mafic rocks [[42], [43], [44]]. Monazite, xenotime, allanite, chevkinite, parisite, zircon, apatite, rutile, columbo-tantalite are the main REEs bearing minerals [37,44]. Except cerium, all other REEs display maximum concentrations in soil samples from this area.
ii) the second area with LREEs anomalies occupies a large surface covering the central, southern and south - western parts of massif. The apex of the LREEs anomaly (Prișica Peak) is associated with metasomatic syenites enriched in REE, U, Th, Zr [69,70]. The main part of the LREEs anomaly (extending towards south-west) is typical for the external zone of massif with soils formed on alkali-syenites, nepheline-syenites, and essexites with gradual transitions among them. In addition, the same LREEs geochemical halo emphasizes the intermediate zone represented by essexites and alkali-syenites cut by nepheline-syenite veins that were hybridized and metasomatically altered. Small REE, Th, Zr, Nb veins and disseminated mineralization occurs in this area [69,70].
iii) the last area located in north - eastern part of massif, where LREEs in soil were generated by the occurrence of monazite, thorite, zircon, aeschynite in granitoids, muscovitized nepheline syenites and liebeneritized syenites [70].

In comparison with LREEs, the spatial distribution of HREEs and yttrium in soils of the massif show only slight anomalies. The distribution maps reveal high levels of HREEs and Y following two trends that can be correlated with the characteristics of parent rocks. The first trend is strictly linked to the low silica content of rocks, with a very good correlation between HREEs and LREEs, remarked in the north-western and central-western parts of massif, associated with veins and disseminated REE mineralization (Jolotca, Ghiduț) in mafic and ultramafic rocks. The second trend is observed in the south-eastern part of massif, being linked to the dominantly Y–Th mineralization associated with the marginal facies syenitic and granitic rocks (quartz-syenite, aplite syenite), as well as similar veins and apophyses developed outside the boundary of the complex [69].

### 5.3 pH of soil and REEs

The analysis of pH values (Tables 1 and S1) shows the dominant frequency of soils with acidic to weakly acidic reaction (pH values ranging from 3.63 to 6.31), and the subordinate frequency of soils with alkaline reaction (pH - 7.38).

**Fig. 10:**
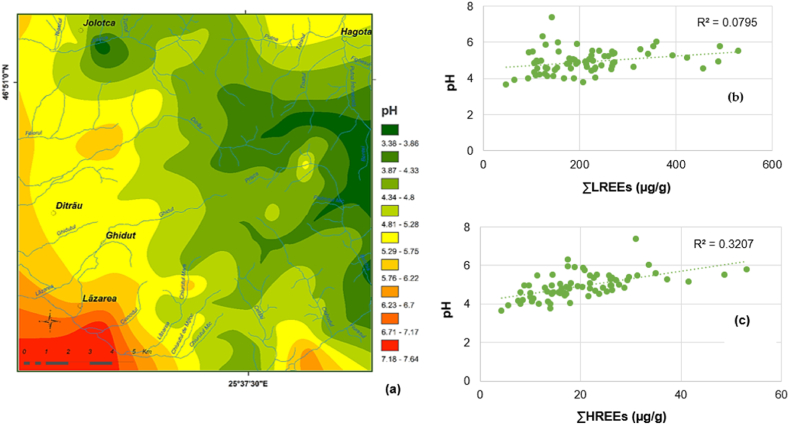
(a) The map of soil pH; (b) Correlation pH – ΣLREEs; (c) Correlation pH – ΣLREEs.

Based on the correlation of the pH map (Fig. 10 (a)) with the soil and geological substrate maps, the following observations can be made:

a. the lowest pH values, ranging from 3.4 to 5.2, characterize leptosols, distric cambisols and eutric cambisols described in the central, central-southeastern, and eastern part of the massif, developed on syenites, nepheline syenites, metasomatic syenites, granitoid rocks and crystalline schists at the massif border zone;
b. A weakly acidic reaction (pH = 5.2–6.6) describes luvisols and gleysols developed on sedimentary formations and fluvial deposits presents in the central – western and western part of the massif;
c. redzic leptosols developed on marble and dolomites show a neutral to weakly alkaline soil reaction;
d. the pH of the soil increases from the eastern to western part of the massif.

Acidic - weakly acidic soils (pH 3.5–6.6) are soils leached from salts, their reaction being determined by the ratio of bases to hydrogen adsorbed in the clay-humic complex; the lower the saturation of the clay-humic complex in bases, the higher the acidity. Since the soils are not saturated in bases, the free carbonic acid, which comes from the dissolution of carbon dioxide in the water, increases the acidity. Mineral acids (phosphoric, nitric, sulphuric), being subject to leaching, are absent or found in small quantities in free form and do not influence the reaction, compared to fulvic acids dissolved in the soil solution which cause a strongly acidic soil reaction [52].

In the case of rendzinas, the soil reaction is determined by the presence of salts that hydrolyze basically, especially carbonates, which are spread all over the soil mass and which, under conditions of natural equilibrium of carbonic acid in the soil solution with carbon dioxide in the air, cause a neutral - weakly alkaline reaction [52].

Hence, under normal conditions, in the absence of strong anomaly-generating sources, all REEs are described as being more concentrated in alkaline soils than in acidic soils, probably due to the slight removal of their hydroxyl complexes [74].

The correlation of pH map (Fig. 10 (a)) with REEs distribution maps (ΣLREEs and ΣHREEs (Fig. 9)) shows that acidic and weakly acidic soils are characterized by intensive accumulations of REEs, especially LREEs, reflecting the specific composition of the rock from which they were derive, and mineralization of REEs in the study area [42].

The correlation plots of pH vs. ΣLREEs (Fig. 10. (b)), respectively, pH vs. ΣHREEs (Fig. 10 (c)) indicate positive correlations given by the two groups of REEs (calculated as Sum) and pH, but very low correlations in case of ΣLREEs (r^2^ = 0.079) (Fig. 10(b)), and low to moderate correlations for ΣHREEs (r^2^ = 0.3227) (Fig. 6 (c)). Although acidic and weakly acidic soils are enriched in LREEs, these data confirm that pH does not control the accumulation of LREEs in soil, and influences the concentration of HREEs only to a small extent, indicating heavy minerals rich in REEs and resistant to weathering as the principal common source for them [10,24].

Both graphs suggest the general trend of REEs mobility in pedogenesis; the mobility of REEs increases with increasing atomic number, heavy rare earth elements being more mobile than light ones. For ΣHREEs, diagram illustrates the tendency of heavy rare earth elements to accumulate in soils with a less alkaline reaction than light rare earth elements.

The data suggest that soil pH is not an important factor in the REEs preferential enrichment of soils from the Ditrău massif area, the REEs concentrations being strongly dependent on the type of REEs bearing minerals in the rocks. Mineralogical studies developed on rocks at Ditrău confirm the presence of REE-bearing minerals (heavy minerals) enriched in LREEs and depleted in HREEs such as monazite, allanite, pyrochlore, columbite, aeschynite, apatite and thorite [[44], [45], [46], [47], [48]].

### 5.4 ΣLREEs/ΣHREEs, (La/Yb)ch, (La/Sm)ch, (Gd/Yb)ch ratios

**Fig. 11:**
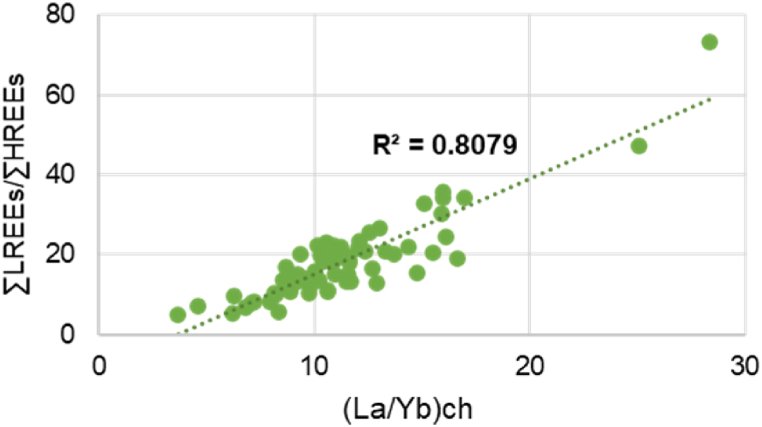
Correlation between ∑LREEs/∑HREEs ratio and (La/Yb)ch in soil samples.

REEs Characteristics (ΣLREEs/ΣHREEs, (La/Yb)ch, (La/Sm)ch, (Gd/Yb)ch ratios) of topsoil samples from the Ditrău Alkaline Massif are listed in Table S4, while statistical parameters (min, max and mean) for REEs Characteristics are found in Table 5. The ratio of ΣLREEs/ΣHREEs varies, regardless of the soil types between 3.71 and 28.37 suggesting, likewise, the preferential enrichment of soils in LREEs and depletion in HREEs. This observation is enforced by the ratio of (La/Yb)ch, that shows a strong correlation with ∑LREE/∑HREE (Fig. 11). Chondrite-normalized (La/Yb)ch ratios in the Ditrău massif soils range between 5.01 and 73.32 with a mean of 18.05, showing significant LREEs fractionation.

**Table 5:** ΣLREEs/ΣHREEs, (La/Yb)ch, (La/Sm)ch, (Gd/Yb)ch ratios and Ce, Eu, Gd, Tb and Ho anomalies.

Variable	LREE/HREE	δCe	δEu	δGd	δTb	δHo	(La/Yb)_ch_	(La/Sm)_ch_	(Gd/Yb)_ch_
Mean	10.65	0.73	0.75	1.08	1.01	0.86	18.05	5.93	1.80
Max.	28.37	1.46	1.14	2.04	1.64	1.34	73.32	16.86	3.96
Min.	3.71	0.48	0.42	0.69	0.43	0.57	5.01	2.66	0.72

The (La/Sm)ch in all soil samples was found higher than 1, ranging from 2.66 to 16.86, and indicating the relative fractionation for middle rare earth elements (Sm, Eu). The HREEs fractionation is almost negligible, being expressed by (Gd/Yb)ch ratio. The (Gd/Yb)ch ratio is mostly positive (>1) the majority of the data ranging from 0.72 to 3.96. The high value of (La/Yb)ch, (La/Sm)ch, (Gd/Yb)ch ratios are typical for soils associated with metasomatic syenites (Peak Prişica zone) that are strongly enriched in cerium and lanthanum and strongly depleted in the other REEs. The lowest (La/Yb)ch (La/Sm)ch, (Gd/Yb)ch values is characteristic for soils formed on metamorphic rocks (schists) beyond the southern boundary of the massif. The individual (La/Yb)ch, (La/Sm)ch, (Gd/Yb)ch ratios express the degree of REEs that would confirm a LREEs enrichment compared with and HREEs in soils developed on alkaline rocks [71].

### 5.5 The chondrite-normalized REEs patterns

**Fig. 12:**
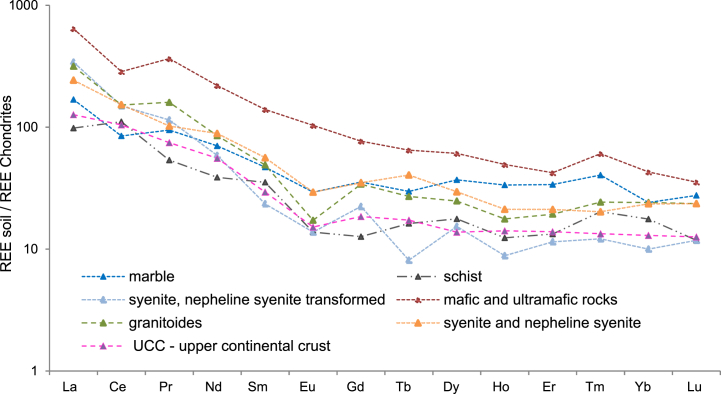
Chondrite – normalized REEs patterns for the soil samples [64].

The chondrite-normalized REEs patterns (Fig. 12) also generally correlate with the nature of the soil substratum, with fractionation of the LREEs, values similar to the average UCC for metamorphic rocks and transformed syenite, and enrichment from acid to basic rocks. A negative Eu anomaly appears in soils developed on syenites, nepheline sienites, and granitic rocks.

The values of Ce, Eu, Gd, Tb and Ho anomalies are summarized in Table 5 and detailed in Table S 4. Cerium anomaly values (δCe) vary from 0.48 to 1.46 and the negative Ce-anomaly was recorded in the most mature soils developed on basic rocks, hornfelses, transformed syenites, granites and crystalline limestones, whereas the cerium positive anomaly was identified in soil samples developed on schists, syenites and nepheline syenites. The cerium anomaly reflects the solubility of this element in tetravalent state under environmental condition during weathering, that produces the oxidation of Ce^3+^ to Ce^4+^ [83].

The europium anomaly varies from 0.72 to 1.14. Except for soils formed on mafic and ultramafic rocks, different degrees of negative Eu anomaly were observed in all other soils samples from the Ditrău massif. The bedrocks (granite, syenite, schist) are characterized by negative Eu anomalies generated by plagioclase fractionation (Eu^2+^ substituting Ca^2+^) in the course of rock forming processes [84]. During weathering, Eu^2+^ is oxidized to Eu^3+^ and is released from plagioclease, behaving like all other REEs. Most of the negative Eu-anomaly values in soil samples studied are similar to those of the parent rocks [42]. The europium anomalies from soils developed on mafic and ultramafic rocks have values close to the one mimicking the typical situation of basic rocks.

A significant number of soil samples from the Ditrău massif show positive Gd anomalies (δGd values from 0.69 to 2.04). The soils covering the mineralized veins Jolotca and Belcina (characterized by high Gd content) and metamorphic rocks show distinct positive gadolinium anomalies. The Tb-anomaly has values which range from 0.43 to 1.64 displaying a good correlation with the Gd anomalies (0.51) suggesting that both mentioned anomalies were generated by the same process. In addition, the majority of the soil samples show a negative anomaly for ytterbium (0.67–1.89) and holmium (δ Ho values: 0.57–1.34), with an unclear explanation.

**Fig. 13:**
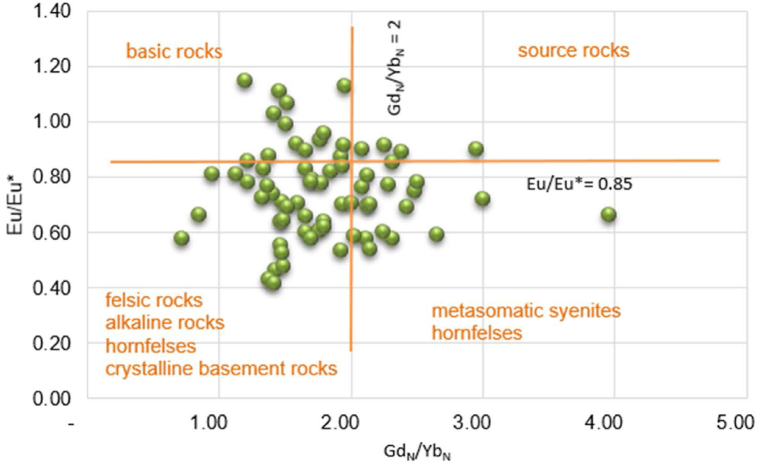
The Eu/Eu* versus Gd_N_/Yb_N_ plot in soil samples.

Although is typical for estimation of the amount of Eu depletion and HREE depletion fractionation over time in sediments, a plot of Eu/Eu* versus Gd_N_/Yb_N_ (Fig. 13) was used to identify detrital material sources in investigated soils. Upper crustal rocks typically contain Gd_N_/Yb_N_ ratios ranging from 1.0 to 2.0, however these ratios can be affected by heavy mineral concentrations [84,85]. In the Ditrău soils, the Eu/Eu* and Gd_N_/Yb_N_ ratios range from 0.42 to 1.14 (avg. 0.75) and 0.72 to 3.96 (avg. 1.80) respectively indicating that the detrital material is derived from alkaline rocks (syenites, nepheline syenites), felsic rocks (granites) and basic rocks (mafic and ultramafic rocks) (Fig. 13). Also, the Eu/Eu* vs (Gd/Yb)_N_ plot indicates that in soil samples plotted in right field of the crossing Eu/Eu* vs (Gd/Yb)_N_ the content of heavy minerals in the soil mass is higher [84]. The soil samples plotted in this field with Gd_N_/Yb_N_ ratios up to 3.96 and negative anomaly for europium are derived by essexites ((hybrid alkaline gabbro, diorite and monzonite, veined and metasomatically altered by nepheline syenites) described as enriched in monazite, thorite, zircon [69.70].

### 5.6 Correlation analysis

Pearson's correlation was applied to establish potency associations and linear relations between elements form rare earth elements (REEs) group. Furthermore, the Pearson's analysis allowed the assessment of relationships between each element of rare earth elements group and pH values. As can be reported in Table 6, all REEs elements, yttrium and pH values exhibit a linear correlation matrix.

**Table 6:** Pearson correlation among selected REEs, Y and pH in soils of the study area (N = 70).

	La	Ce	Pr	Nd	Sm	Eu	Gd	Tb	Dy	Ho	Er	Tm	Yb	Lu	Y	pH
La	1															
Ce	0.873 0.873	1														
Pr	0.889	0.737	1													
Nd	0.820	0.770	0.954	1												
Smm	0.723	0.625	0.923	0.944	1											
Eu	0.717	0.597	0.905	0.913	0.926	1										
Gd	0.716	0.677	0.885	0.937	0.918	0.905	1									
Tb	0.615	0.588	0.812	0.884	0.903	0.875	0.929	1								
Dy	0.618	0.547	0.821	0.862	0.885	0.886	0.916	0.917	1							
Ho	0.536	0.489	0.750	0.810	0.834	0.848	0.877	0.879	0.951	1						
Er	0.529	0.514	0.715	0.784	0.809	0.786	0.860	0.858	0.935	0.935	1					
Tm	0.430	0.387	0.643	0.685	0.744	0.731	0.791	0.789	0.873	0.890	0.896	1				
Yb	0.493	0.449	0.687	0.723	0.786	0.771	0.789	0.824	0.880	0.845	0.891	0.864	1			
Lu	0.374	0.407	0.551	0.629	0.657	0.628	0.716	0.759	0.773	0.794	0.805	0.790	0.790	1		
Y	0.571	0.494	0.761	0.806	0.840	0.855	0.889	0.884	0.955	0.957	0.939	0.901	0.887	0.823	1	
pH	0.211	0.238	0.282	0.374	0.413	0.403	0.520	0.490	0.542	0.563	0.608	0.598	0.506	0.533	0.640	1

All REEs in soils of Ditrău massif area are very strongly correlated among themselves. The most correlation coefficients are higher than 0.8, and reflects the geochemical similarity between them (have similar chemical and physical properties). Also, the correlation reveals the natural common sources of these elements. REEs do not occur separately in nature, they are present together in a variety of ore/accessory minerals as minor or major components, typically represented by heavy minerals such as: monazite, xenotime, zircon, rutile and columbo-tantalite) [86]. The highest correlation coefficients are 0.95 for Nd - Pr, and 0.95 for Ho - Dy. These adjacent pairs of elements constitute the correlation nucleus for the two groups of REEs: the light rare elements or ceric group LREEs - La, Ce, Pr, Nd, Sm and Eu; and the heavy rare earth elements or yttric group HREEs - Gd, Tb, Dy, Ho, Er, Tm, Yb, and Lu. The strongly correlations between neighboring elements are given by the more similarity in atomic weight and ionic radius.

Yttrium has a very strong correlation (>0.8) with all HREEs (from Dy to Lu), a strong correlation (>0.7) with Eu, Sm, Nd, Pr among LREEs and a good correlation with cerium (0.49) and lanthanum (0.57). The highest correlation coefficients are displayed with Dy (0.95) and Ho (0.95), and reflect the yttrium affinity for heavy rare earth elements generated by their similar geochemical behavior.

All these correlations suggested that the bulk distribution of REEs in soils of the Ditrău massif is similar to that of the heavy minerals in the bedrock [42] and the most important part of HREEs are incorporated together with LREEs in these minerals (monazite, xenotime, thorite, pyrochlore, columbite, fergusonite, euxenite, aeschynite, zircon, titanite, garnet), all of which are resistant to weathering. The strong correlation of cerium (0.87) with lanthanum indicates monazite as the main carrier of REEs in soil. Another phase abundant in some mineralized veins, xenotime, seems to be responsible for local high Y contents and good correlations between this element and HREEs. Probably, a small fraction the HREEs and yttrium in soils, according to Ref. [77], is generated by the dissolution of xenotime. Yttrium also may display good correlations with LREEs when the source rocks are described as rich in monazite [69.70].

The Pearson's analysis allowed the assessment of relationships between each element of rare earth elements group and pH values. The correlation coefficients indicate an increase of the coefficients from LREEs to HREEs and Y. The LREEs have values of coefficients between 0.21 and 0.40, whereas HREEs, and Y display values above 0.5. This trend indicates that pH is less important in the accumulation of individual REEs and Y in soil, implying lesser mobility of LREEs under pH conditions and more availability of HREEs for dissolution, complexation, and adsorption.

## 6 Conclusions

The topsoils from Ditrău Alkaline Massif area are enriched in LREEs and relative depleted in HREEs and yttrium. The mean of individual contents of REEs indicates the following geochemical abundance order: Ce > La > Nd > Pr > Sm > Gd > Dy > Yb > Er > Eu > Ho > Tb > Tm > Lu. Individual REEs contents are higher than those in the earth's crust and similar environments. Ce, La and Nd are the most abundant REEs in soils, with concentrations up to 235.70 μg/g, 181.1 μg/g and 106.80 μg/g respectively. This pattern of REEs abundance in soil is similar to that described in the parent rocks, reflecting the degree of enrichment in these rocks. The variation of ΣLREEs/ΣHREEs and (La/Yb)ch ratios indicates preferential enrichment of soils in LREEs and depletion in HREEs.

The parent material was the main factor that controlled distribution and abundance of REEs in topsoils of Ditrău Alkaline Massif area, independent of soil types, acidity of soil and weathering degree. The spatial distribution of REEs in Ditrău Alkaline Massif area revealed that the prevalence of REEs is controlled by the mineralogical composition of the parental materials. Over the entire surface of the Ditrău massif, the high concentrations of REEs in soils are associated with REEs mineralization and the presence of minerals refractory to weathering (monazite, zircon, titanite, allanite, rutile, garnet, apatite, columbo-tantalite, a.s.o.), thus explaining the pattern of correlation. The pH of soils is generally acidic to weakly acidic and has an insignificant role in REEs fractionations in soil.

The distribution of REEs in topsoils of the Ditrău area is an almost unchanged reflection of that of the parental rocks and represents the typical situation in thin young soils with undeveloped soil profile, rich in skeletal material represented by unaltered rock fragments, formed in temperate-continental forest (mostly needleleaf) areas, unaffected by the anthropic factor. This context and the presence of REE mineralizations in the bedrock substratum enables REE distribution in soils as a valuable tool in detecting and tracing mineralized zones in the parent rocks.

## Author contribution statement

Conceived and designed the experiments.

Performed the experiments.

Analyzed and interpreted the data.

Contributed reagents, materials, analysis tools or data.

Wrote the paper.

## Funding statement

This work was supported by Romanian Ministry of Research, Innovation and Digitalization [PN-09.21.01.03/2014, PN-19.45.02.03/28N/2019 and PN 23-39.02.03/34N/2023].

## Data availability statement

Data included in article/supplementary material/referenced in article.

## Declaration of interest’s statement

The authors declare no conflict of interest.

## References

[1] Haneklaus Hu Z., Sparovek S., Schnug G.E. (2006). Rare earth elements in soils. Commun. Soil Sci. Plant Anal..

[2] Marshall C.P., Fairbridge R.W., S. M., McLennan, Yttrium (1999). Encyclopedia of Geochemistry. Encyclopedia of Earth Science.

[3] Cao X., Chen Y., Wang X., Deng X. (2001). Effects of redox potential and pH value on the release of rare earth elements from soil. Chemosphere.

[4] Aide M.T., Aide C. (2012). Rare earth elements: their importance in understanding soil genesis, international scholarly research network. ISRN Soil Sci..

[5] Taylor S.R., McLennan S.M. (1985).

[6] Dupré B., Gaillardet J., Rousseau D. (1996). Major and trace elements of river-borne material: the Congo Basin. Geochem. Cosmochim. Acta.

[7] Gaillardet J., Viers J., Dupré B., Holland H.D., Turekian K.K. (2004).

[8] L Rudnick R., Gao S., Holland H.D., Turekian K.K. (2003).

[9] Ramesh E., Ramesh P.S. (2000). Distribution of rare earth elements and heavy metals in the surficial sediments of the Himalaya River system. Geochem. J..

[10] Laveuf C., Cornu S., Juillot F. (2008). Rare earth elements as tracers of pedogenetic processes. Compt. Rendus Geosci..

[11] Sadeghi M., Morris G.A., Carranza E.J.M., Ladenberger A., Andersson M. (2013). Rare earth element distribution and mineralization in Sweden: an application of principal component analysis to FOREGS soil geochemistry. J. Geochem. Explor..

[12] Petrosino P., Sadeghi M., Albanese S., Andersson M., Lima A., De Vivo B. (2013). REE contents in solid sample media and stream water from different geological contexts: comparison between Italy and Sweden. J. Geochem. Explor..

[13] Ren L., Cohen D.R., Rutherford N.F., Zissimos A.M., Morisseau E.G. (2015). Reflections of the geological characteristics of Cyprus in soil rare earth element patterns. Appl. Geochem..

[14] Silva C., Barbosa R.S., Do Nascimento C., Yjab S. (2018). Geochemistry and spatial variability of rare earth elements in soils under different geological and climate patterns of the Brazilian Northeast. Rev. Bras. Ciência do Solo.

[15] Sadeghi M., Petrosino P., Ladenberger A., Albanese S., Andersson M., Morris G., Lima A., De B., Vivo Ce (2013). La and Y concentrations in agricultural and grazing-land soils of Europe. J. Geochem. Explor..

[16] Henderson P., Henderson P. (1984). Developments in Geochemistry 2.

[17] Ran Y., Liu Z. (1992). Adsorption and desorption characteristics of rare earth elements in main soil types in China. Chin. Sci. Bull..

[18] Foley N., Ayuso R., Simandl G.J., Neetz M. (2015).

[19] Shan X.Q., Lian J., Wen B. (2002). Effect of organic acids on adsorption and desortion of rare earth elements. Chemosfere.

[20] Pourret O., Davranche M., Gruau G., Dia A. (2007). Experimental rare earth elements complexation with humic acid. Chem. Geol..

[21] Ding S.M., Liang T., Zhang C.S. (2005). Accumulation and fractionation of rare earth elements (REEs) in wheat: controlled by phosphate precipitation, cell wall absorption and solution complexation. J. Exp. Bot..

[22] Zhou W., Han G., Liu M., Song C., Li X. (2020). Geochemical distribution characteristics of rare earth elements in different soil profiles in mun river basin, northeast Thailand. Sustainability.

[23] Johannesson K.H., Lyons W.B., Stetzenbach K.J., Byrne R.H. (1995). The solubility control of rare earth elements in natural terrestrial waters and the significance of PO_4_^3-^ and CO_3_^2-^ in limiting dissolved rare earth concentrations: a review of recent information. Aquat. Geochem..

[24] Laveuf C., Cornu S., Guilherme L.G.R., Guerin A., Juillot F. (2012). The impact of redox conditions on the rare earth element signature of redoximorphic features in a soil sequence developed from limestone. Geoderma.

[25] Foley N.K. (2013). Rare earth elements: the role of geology, exploration, and analytical geochemistry in ensuring diverse sources of supply and a globally sustainable resource. J. Geochem. Explor..

[26] Balintoni I. (1981). The importance of the Ditrau alkaline massif emplacement moment for the dating of the basement overhrusts in the East Carpathians. Rev. Roum. Geol. Geophys. Geograph. Ser. Geol..

[27] Mureșan M. (1983).

[28] Kräutner H.G., Bindea G. (1998). Timing of the Ditrău alkaline intrusive complex (eastern charpatians, Romania). Slovak Geol. Mag..

[29] Pál-Molnár E., Szakall S., Kristaly F. (2010). Mineralogy of Székeland Eastern Transilvania.

[30] Pál-Molnár E., Kirikiri L., Lukács R., Dunkl I., Batki A., Szemerédi M., Eszter Almási E., Sogrik E., Harangi S. (2021). Timing of magmatism of the Ditrău Alkaline Massif, Romania – a review based on new U–Pb and K/Ar data. Centr. Eur. Geol..

[31] Dallmeyer R.D., Kräutner H.G., Neubauer F. (1997). Middle - late Triassic ^40^Ar/^39^Ar hornblende ages for early intrusion within the Ditrău Alkaline Massif, Romania: implication for Alpine rifting in the Carpathian orogen. Geol. Carpathica.

[32] Pană D., Balintoni I., Heaman L. (2000). The U-Pb zircon dating of the syenite phase from the Ditrau alkaline igneous complex. Studia UBB Geologia.

[33] Morogan V., Upton B.G.J., Fitton J.G. (2000). The petrology of the Ditrău alkaline complex, Eastern Carpathian. Min. Pet..

[34] Codarcea A., Ianovici V., Iova I., Papacostea C. (1958). Elemente rare în masivul de la Ditrău, Com. Acad. R.P.Rom..

[35] Streckeisen A., Hunziker J.C. (1974). On the origin and age of the nepheline-syenite complex of Ditró (Transylvania, Rumania). Schweizer Mineralogische Mitteilunge.

[36] Atanasiu N., Constantinescu E. (1984). Contributions á la connaissance pétrologique et structurale du massif alcalin de Dirtău. Analele Universităţii Bucureşti.

[37] Anastasiu N., Garbasevschi N., Jakab G., Vlad S., Borcos M., Vlad S. (1994). IGCP Project 356, Plate Tectonics and Metallogeny in the East Carpathians and Apuseni Mts. (Field Trip Guide).

[38] Pál-Molnár E. (2000). Geochemistry and Petrology.

[39] Pál-Molnár E., Batki A., Almási E., Kiss B., Upton B.G., Markl G., Odling N., Harangi S. (2015). Origin of mafic and ultramafic cumulates from the Ditrău alkaline massif, Romania. Lithos.

[40] Pál-Molnár E., Batki A., Ódri Á., Kiss B., Almasi E. (2015). Geochemical implications for the magma origin of granitic rocks from the Ditrău alkaline massif (eastern Carpathians, Romania). Geol. Croat..

[41] Streckeisen A. (1960).

[42] Jakab G. (1998).

[43] Honour V.C., Goodenough K.M., Shaw R.A., Gabudianu I., Hîrtopanu P. (2018). REE mineralisation within the Ditrău Alkaline Complex, Romania: interplay of magmatic and hydrothermal processes. Lithos.

[44] Săbău G., Anastasiu N., Duliu O. (2009). Mineralogy and Geodiversity - Tributes to the Career of Professor Emil Constantinescu.

[45] Hîrtopanu P., Andersen J., Fairhurst R., Nb Ta, Ree ( Y. (2010).

[46] Hîrtopanu P., Andersen J., Fairhurst R., Jakab G. (2013).

[47] Hîrtopanu P., Jakab G., Andersen C., Fairhurst J. (2013).

[48] Hîrtopanu P., Fairhurst R.J., Jakab G., Andersen C. (2015).

[49] Florea N., Munteanu I. (2003).

[50] Țărănu D., Rogobete Gh, Dicu D., Nita L. (2012). Romanian soil taxonomy system SRTS-2012. Agric. Sci. Technol..

[51] Micheli E., Schad P., Spaargaren O., WRB (2006).

[52] Donisă D., Rusu C., Mocanu R., Lupașcu A. (1998). Aspecte fizico-chimice cu implicații pedogenetice ale andosolurilor şi solurilor adiacente din Munții Oaș-Igniș, Lucrările Seminarului Geografic Dimitrie Cantemir. Univ. Al. I. Cuza Iași.

[53] Reimann C., Albanese S., Batista M.J., Bel-Lan A., Birke M., Cicchella D., Demetriades A., De Vivo B., De Vos W., Dinelli E., Duris M., Dusza-Dobek A., Ernstsen V., Flight D., Gilucis A., Gosar M., Gregorauskiene V., Gulan A., Hayoz P., Halamic J., Haslinger E., Hrvatovic H., Ion A., Ivanovna Y., Johnson C., Jordan G., Kisivilla J., Klein P., Kwecko P., Lax K., Lima A., Locutura J., Malyuk B.I., Maquil R., Marku S., Martins L., Mazreku A., Messina A., O’connor P., Ottesen R.T., Pasieczna A., Petersell W., Reeder S., Salpeteur I., Schedl A., Sefcik P., Slaninka I., Sorsa A., Selinus O., Stafilov T., Tarvainen T., Trendavilov V., Utermann J., Valera P., Vidojevic D., Volden T. (2008).

[54] Salminen R., Batista M.J., Bidovec M., Demetriades A., De Vivo B., De Vos W., Duris M., Gilucis A., Gregorauskiene V., Halamić J., Heitzmann P., Jordang Klaver G., Klein P., Lis J., Locutura J., Marsina K., Mazreku A., O’connor P.J., Olsson S.A., Ottesen R.T., Petersell V., Plant J.A., Reeder S., Salpeteur I., Sandström H., Siewers U., Steenfelt A., Tarvainen T. (2005).

[55] Fabian C., Reimann C., Fabian K., Birke M., Baritz R., Haslinger E. (2014). GEMAS: spatial distribution of the pH of European agricultural and grazing land soil. Appl. Geochem..

[56] Reimann C., Filzmoser P., Garrett R.G. (2005). Background and threshold: critical comparison of methods of determination. Sci. Total Environ..

[57] Huang H., Lin C., Yu R., Yan Y., Hu G., Wang Q. (2019). Spatial distribution and source appointment of rare earth elements in paddy soils of Jiulong River Basin. J. Geochem. Explor..

[58] Pourmand A., Dauphas N., J Ireland T. (2012). A novel extraction chromatography and MC-ICP-MS technique for rapid analysis of REE, Sc and Y: revising CI-chondrite and Post-Archean Australian Shale (PAAS) abundances. Chem. Geol..

[59] S Ferreira M.D., Fontes M.P.F., Bellato Marques C.R., Neto J.O., Lima H.N., Fendorf S. (2021). Geochemical signatures and natural background values of rare earth elements in soils of Brazilian Amazon. Environ. Pollut..

[60] Sutherland R.A. (2000). Bed sediment-associated trace metals in an urban stream, Oahu, Hawaii. Environ. Geol..

[61] Muller G. (1969). Index of Igeo accumulation in sediments of the rhine river. Geojournal.

[62] Muller G. (1979). Schwermetalle in den sedimenten desrheins veranderungenseit. Umschau.

[63] Muller G. (1981). The heavy metal pollution of the sediment of Neckars and its tributary: astocktaking. Chemistry in Our Time.

[64] Sun Shen-su, McDonough W.F., D Saunders A., Norry M.J. (1989).

[65] Lin C., Liu S., He M., Li R. (2013). Distribution of rare earth elements in the estuarine mand coastal sediments of the Daliao River System, China. J. Radioanal. Nucl. Chem..

[66] De Baar H.J.W., Bacon P., Brewer P.G., Bruland K.W. (1985). Rare earth elements in the atlantic and pacific oceans. Geochem. Cosmochim. Acta.

[67] Kato Y., Nakao K., Isozaki Y. (2002). Geochemistry of Late Permian Triassic pelagic cherts from southwest Japan: implications for an oceanic redox change. Chem. Geol..

[68] Karanlık S., Ağca N., Yalçın M. (2011). Spatial distribution of heavy metals content in soils of Amik Plain (Hatay, Turkey). Environ. Monit. Assess..

[69] Zincenco D., Vlad S., Grabari G. (1981).

[70] Zincenco D., Petrescu M., Popescu C., Prodanescu I., Zincenco C. (1994). Age and petrology of the Ditrau massif: rocks of the enveloping facies, Report. Arh. Soc. Prosp. Bucureşti.

[71] Chen J., Yang R. (2010). Analysis on REE geochemical characteristics of three types of REE-rich soil in Guizhou Province, China. J. Rare Earths.

[72] de Sá Paye H., de Mello J.W.V., de Magalhães Mascarenhas G.R.L., Gasparon M. (2016). Distribution and fractionation of the rare earth elements in Brazilian soils. J. Geochem. Explor..

[73] Bispo F.H.A., de Menezes M.D., Fontana A., Sarkis J.E.d.S., Gonçalves C.M., de Carvalho T.S., Curi N., Guilherme L.R.G. (2021). Rare earth elements (REEs): geochemical patterns and contamination aspects in Brazilian benchmark soils. Environ. Pollut..

[74] Kabata-Pendias A. (2001).

[75] T Liu Q., Diamond M.E., Gingrich S.E., Ondov J.M., Maciejczyk P., Sterm G.A. (2003). Accumulation of metals, trace elements and semivolatile organic compounds on exterior windows surfaces in Baltimore. Environ. Pollut..

[76] Tyler G., Olsson T. (2002). Conditions related to solubility of rare and minor elements in forest soils. J. Plant Nutr. Soil Sci..

[77] Tyler G. (2004). Rare earth elements in soil and plant systems - a review. Plant Soil.

[78] Hoshino M., Sanematsu K., Watanabe Y. (2016). REE mineralogy and resources. Handb. Phys. Chem. Rare Earths.

[79] Temga J.P., Sababa E., Mamdem L.E., Bijeck M.L.N., Azinwi P.T., Tehna N., Zo’o Zamea P.Z., Onana V.L., Nguetnkam J.P., Bitom L.D., Ndjiguia P.D. (2021). Rare earth elements in tropical soils, Cameroon soils (Central Africa). Geoderma Reg..

[80] Cao X., Wu P., Cao Z. (2016). Element geochemical characteristics of a soil profile developed on dolostone in central Guizhou, southern China: implications for parent materials. Acta Geochim..

[81] Alfaro M.R., Do Nascimento C.W.A., Biondi C.M., Agra Bezerra da Silva Y.J., Agra Bezerra da Silva Y.J., De Aguiar Accioly A.M., Montero A., Ugarte O.M., Estevez J. (2018). Rare-earth-element geochemistry in soils developed in different geological settings of Cuba. Catena.

[82] Faris N., Ram R., Tardio J., Bhargava S., Pownceby M.I. (2019). Characterization of a ferruginous rare earth bearing lateritic ore and implications for rare earth mineral processing. Miner. Eng..

[83] Matini L., Ossebi J.G., Mbedi R., Moutou J.M. (2012). Rare earth elements in soil on spoil heap of an abandoned lead ore treatment plant in the district of mfouati, Congo-brazzaville. I. Res. J. Environ. Sci..

[84] McLennan S.M. (1989). Rare earth elements in sedimentary rocks: influence of the provenance and sedimentary process. Rev. Mineral. Geochem..

[85] McLennan S.M. (1993). Weathering and global denudation. J. Geol..

[86] Dostal J. (2017). Rare earth element deposits of alkaline igneous rocks. Resources.

