# Supplementary material for: Rare earth elements distribution in topsoil from Ditrău Alkaline Massif area, eastern Carpathians, Romania
Source: Heliyon. 2023 Feb 24;9(3):e13976. doi: 10.1016/j.heliyon.2023.e13976 (PMC10006540; doi:10.1016/j.heliyon.2023.e13976)
Supplement: Multimedia component 1 [file mmc1.docx]

**Supplementary Data**

**Table S1**

Concentration of REEs (μg/g) and pH values in the topsoils of Ditrău Alkaline Massif

| ***Sample***  ***ID*** | ***La*** | ***Ce*** | ***Pr*** | ***Nd*** | ***Sm*** | ***Eu*** | ***Gd*** | ***Tb*** | ***Dy*** | ***Ho*** | ***Er*** | ***Tm*** | ***Yb*** | ***Lu*** | ***Y*** | ***pH*** |
| --- | --- | --- | --- | --- | --- | --- | --- | --- | --- | --- | --- | --- | --- | --- | --- | --- |
| 1 | 40 | 51.9 | 9 | 32.8 | 7.2 | 1.7 | 7.3 | 1.1 | 9.4 | 1.9 | 5.6 | 1 | 4.1 | 0.7 | 48.1 | 7.38 |
| 2 | 36.3 | 78.4 | 8 | 26.4 | 5.2 | 1.1 | 5.2 | 0.8 | 4.6 | 0.9 | 2.7 | 0.4 | 2.4 | 0.5 | 18.6 | 5.95 |
| 3 | 119 | 158.1 | 15.8 | 52.6 | 7.1 | 1.7 | 6.3 | 1 | 6.4 | 0.9 | 3.1 | 0.4 | 2.5 | 0.3 | 22.3 | 5.75 |
| 4 | 52.1 | 86.1 | 10.2 | 37.3 | 8 | 1.4 | 6.1 | 0.8 | 5.8 | 0.9 | 2.8 | 0.3 | 1.9 | 0.3 | 16.8 | 5.9 |
| 5 | 33.2 | 54.4 | 5.8 | 17.2 | 3.4 | 0.5 | 3.1 | 0.4 | 2.6 | 0.4 | 1.1 | 0.2 | 1.8 | 0.2 | 8.8 | 4.93 |
| 6 | 81.7 | 138 | 17.4 | 63.8 | 9.6 | 2.9 | 10 | 1.3 | 6.1 | 1.4 | 2.9 | 0.4 | 2.8 | 0.4 | 25.9 | 4.63 |
| 7 | 105.6 | 160.9 | 18.5 | 58.8 | 12.8 | 3.1 | 11.3 | 1.6 | 9.3 | 1.6 | 4.6 | 0.8 | 4.1 | 0.3 | 36.6 | 6.02 |
| 8 | 49.7 | 91.6 | 11.2 | 36.3 | 6.8 | 1.7 | 5.4 | 1 | 4 | 0.8 | 2.4 | 0.6 | 2.7 | 0.3 | 18.4 | 4.87 |
| 9 | 39.6 | 69.7 | 9 | 29.6 | 8.8 | 1.4 | 6.2 | 1 | 5.4 | 0.9 | 4 | 0.6 | 3.5 | 0.4 | 23.6 | 5.47 |
| 10 | 45.6 | 84.2 | 10.1 | 30.4 | 7.4 | 1.3 | 5.4 | 0.8 | 5.3 | 0.7 | 2.6 | 0.4 | 2.7 | 0.3 | 17.9 | 5.08 |
| 11 | 41.8 | 83.8 | 10.4 | 34.1 | 7.5 | 1.5 | 6 | 1.3 | 8.6 | 1.1 | 4.8 | 0.6 | 5.9 | 0.5 | 27.4 | 4.83 |
| 12 | 60.8 | 95.7 | 12.5 | 48.1 | 8.8 | 1.6 | 7.9 | 1.2 | 5.6 | 0.9 | 3.8 | 0.5 | 3.1 | 0.4 | 24.1 | 5.5 |
| 13 | 25.6 | 49.2 | 6 | 21.1 | 4.4 | 0.8 | 3 | 0.6 | 2.6 | 0.5 | 1.3 | 0.2 | 1.7 | 0.2 | 11.1 | 4.34 |
| 14 | 47.1 | 80.4 | 11.2 | 39.5 | 8 | 1.3 | 5.3 | 0.9 | 4.1 | 0.9 | 2.2 | 0.4 | 1.9 | 0.4 | 18 | 5.02 |
| 15 | 63.6 | 111.9 | 14.2 | 58.2 | 10 | 2.2 | 9 | 1.3 | 8.4 | 1.7 | 4.4 | 0.7 | 3.5 | 0.4 | 28.9 | 5.21 |
| 16 | 57 | 98.3 | 12.6 | 49.9 | 9.1 | 2.2 | 8.7 | 1.8 | 7.4 | 1 | 3.4 | 0.5 | 2.9 | 0.5 | 25.1 | 4.95 |
| 17 | 53.1 | 97.4 | 12.2 | 46.9 | 9.1 | 2.4 | 6.9 | 1.2 | 5.9 | 1.2 | 3.6 | 0.4 | 2.4 | 0.4 | 25.9 | 5.06 |
| 18 | 51.9 | 98 | 11.9 | 41.2 | 9 | 2.1 | 5.3 | 1.1 | 7 | 1.1 | 3 | 0.5 | 3.6 | 0.3 | 25.1 | 4.78 |
| 19 | 26.8 | 57.5 | 6.6 | 25.7 | 6.5 | 1.2 | 5.3 | 0.7 | 4.7 | 1 | 2.5 | 0.5 | 2.5 | 0.4 | 21.9 | 6.31 |
| 20 | 37.5 | 97.9 | 9 | 36.6 | 7.6 | 1.8 | 7.9 | 1.2 | 8 | 1.5 | 3.9 | 0.7 | 4.1 | 0.5 | 27.4 | 4.99 |
| 21 | 29 | 71 | 6.8 | 22.2 | 3.9 | 1.1 | 4.2 | 0.7 | 4.1 | 0.9 | 3.5 | 0.5 | 2.6 | 0.4 | 16.4 | 4.54 |
| 22 | 23.3 | 67.9 | 5.1 | 18.1 | 5.4 | 0.8 | 2.6 | 0.6 | 4.5 | 0.7 | 2.2 | 0.5 | 3 | 0.3 | 13.1 | 4.95 |
| 23 | 37.2 | 86 | 9.2 | 34.9 | 6.4 | 1.1 | 4.9 | 1 | 4.5 | 0.9 | 1.8 | 0.4 | 2.4 | 0.5 | 16.7 | 4.77 |
| 24 | 57.6 | 93.8 | 9.7 | 41.5 | 8.6 | 1.7 | 7.2 | 1.5 | 7.5 | 1.2 | 3.5 | 0.5 | 4 | 0.6 | 30.9 | 4.93 |
| 25 | 75.5 | 122.7 | 12.3 | 49.3 | 8.1 | 1.7 | 6.6 | 1.2 | 7.2 | 1.4 | 3.7 | 0.5 | 3.6 | 0.4 | 26.1 | 4.84 |
| 26 | 77.6 | 131 | 11.9 | 38.8 | 5.4 | 1.7 | 4.9 | 0.7 | 5 | 0.8 | 2.5 | 0.3 | 2.7 | 0.3 | 18.9 | 4.51 |
| 27 | 177.2 | 231.1 | 18.2 | 51.4 | 9.1 | 1.9 | 7 | 1.1 | 4.9 | 0.7 | 2.5 | 0.2 | 2.7 | 0.4 | 17.9 | 4.9 |
| 28 | 143.1 | 235.7 | 18.6 | 51.7 | 6.2 | 1.4 | 6.7 | 0.8 | 4.2 | 0.6 | 1.8 | 0.3 | 1.4 | 0.3 | 13.8 | 4.54 |
| 30 | 28.9 | 60.8 | 8 | 30.5 | 4.9 | 1.1 | 3.3 | 0.6 | 2.4 | 0.4 | 1.4 | 0.3 | 1.6 | 0.4 | 8 | 4.09 |
| 31 | 53.8 | 91.9 | 13.4 | 39.7 | 8.8 | 1.1 | 4.5 | 0.8 | 3.5 | 0.4 | 1.8 | 0.3 | 2.5 | 0.3 | 9.9 | 3.77 |
| 32 | 40.2 | 56 | 11.8 | 32.7 | 7.5 | 1.2 | 5.7 | 0.7 | 5 | 0.7 | 2.1 | 0.3 | 2.2 | 0.3 | 17.4 | 4.44 |
| 33 | 52.3 | 67.5 | 12.1 | 40.2 | 9.1 | 1.5 | 5 | 0.8 | 4 | 0.4 | 1.3 | 0.3 | 2.3 | 0.3 | 11.2 | 4.52 |
| 34 | 64.8 | 72.4 | 11.9 | 31.1 | 7.1 | 1.3 | 3.8 | 0.6 | 3.6 | 0.5 | 1.5 | 0.3 | 1.3 | 0.2 | 11 | 4 |
| 35 | 45.21 | 48.3 | 9.1 | 20.7 | 4.2 | 0.9 | 3.5 | 0.5 | 3 | 0.4 | 1.4 | 0.3 | 1.5 | 0.1 | 9.5 | 4.25 |
| 36 | 52 | 56.8 | 8.7 | 22.8 | 5.4 | 1 | 4.9 | 0.8 | 3 | 0.6 | 1.9 | 0.4 | 2 | 0.2 | 12.5 | 4.1 |
| 37 | 87.89 | 94.3 | 13 | 36 | 8 | 1.7 | 5.6 | 0.8 | 2.5 | 0.3 | 1.3 | 0.3 | 3.3 | 0.4 | 20.5 | 5.49 |
| 38 | 44.41 | 63.4 | 6.2 | 14 | 1.7 | 0.6 | 2.9 | 0.3 | 1.9 | 0.3 | 1 | 0.2 | 1.3 | 0.2 | 10.2 | 4.58 |
| 39 | 39.81 | 44.5 | 7.3 | 15.4 | 2.9 | 0.6 | 2.6 | 0.2 | 1.9 | 0.3 | 1 | 0.2 | 1.3 | 0.2 | 6.8 | 4.15 |
| 40 | 81.01 | 91.6 | 10.9 | 27.4 | 3.6 | 0.8 | 4.6 | 0.3 | 3.9 | 0.5 | 1.9 | 0.3 | 1.7 | 0.3 | 12.5 | 4.63 |
| 41 | 39.37 | 48.2 | 6.8 | 19.6 | 3.2 | 0.5 | 2.5 | 0.3 | 2.5 | 0.4 | 1.2 | 0.2 | 1.4 | 0.1 | 7.4 | 4.2 |
| 42 | 31.21 | 41.5 | 4.8 | 13.2 | 2.6 | 0.6 | 2.4 | 0.4 | 1.9 | 0.3 | 1.3 | 0.1 | 1.5 | 0.2 | 7.5 | 3.99 |
| 43 | 92.74 | 83.2 | 15 | 34.2 | 7.2 | 1.9 | 5.8 | 1 | 5.5 | 0.7 | 2 | 0.3 | 2.5 | 0.2 | 17.6 | 4.02 |
| 44 | 55.48 | 67.9 | 9.6 | 29.5 | 5.1 | 1.4 | 4 | 0.8 | 4.8 | 0.7 | 1.4 | 0.3 | 1.7 | 0.3 | 15.8 | 4 |
| 45 | 24.6 | 25.3 | 3.6 | 9.3 | 1.9 | 0.5 | 1.5 | 0.3 | 1.6 | 0.4 | 0.7 | 0.2 | 0.9 | 0.1 | 6.6 | 3.92 |
| 46 | 67.36 | 72.5 | 11.8 | 33.5 | 7.8 | 1.6 | 4.3 | 0.8 | 6.2 | 0.9 | 2.2 | 0.5 | 2.6 | 0.3 | 22.5 | 4.77 |
| 47 | 48.28 | 59.4 | 10.8 | 29.3 | 5.4 | 1.8 | 3.9 | 0.8 | 4.9 | 0.7 | 2 | 0.4 | 2.7 | 0.3 | 19.3 | 4.58 |
| 48 | 77.76 | 81.1 | 16.7 | 38.3 | 8.7 | 3.1 | 7.8 | 1.1 | 8.3 | 1.2 | 3.2 | 0.7 | 3.3 | 0.4 | 30.4 | 5.24 |
| 49 | 37.49 | 48.3 | 8.7 | 27.3 | 7 | 1.2 | 6.5 | 1.6 | 5.4 | 1.1 | 2.3 | 0.6 | 2.8 | 0.3 | 19.2 | 5.86 |
| 50 | 79.94 | 86.5 | 19.1 | 50.5 | 12.5 | 3.3 | 8.3 | 1.6 | 7.8 | 1.4 | 3.2 | 0.5 | 3.9 | 0.5 | 29.8 | 5.21 |
| 52 | 89.57 | 91.4 | 19.1 | 58.3 | 11.5 | 3.2 | 9.2 | 1.3 | 8.1 | 1.2 | 4 | 0.7 | 4.8 | 0.4 | 28.7 | 5.38 |
| 53 | 34.8 | 36.1 | 6.6 | 20.6 | 4 | 1.2 | 2.9 | 0.4 | 4.1 | 0.5 | 1.5 | 0.2 | 1.7 | 0.2 | 11.7 | 5.45 |
| 56 | 75.97 | 95.6 | 19.5 | 63.5 | 10.4 | 3.2 | 10.9 | 1.6 | 9.1 | 1.3 | 3.5 | 0.6 | 4 | 0.4 | 33.6 | 5.46 |
| 59 | 14.49 | 18.1 | 3.5 | 9.5 | 2.2 | 0.5 | 1 | 0.2 | 1.4 | 0.3 | 0.7 | 0.1 | 0.5 | 0.1 | 5.5 | 3.63 |
| 60 | 84.15 | 87.1 | 17.8 | 45.5 | 10 | 2.2 | 6.6 | 1 | 7.6 | 1.6 | 4.1 | 0.6 | 4.5 | 0.4 | 30.9 | 4.69 |
| 60.1 | 69.06 | 78.1 | 16.9 | 49.4 | 10 | 2.1 | 5.8 | 1.2 | 6.8 | 1.3 | 3.1 | 0.4 | 2.7 | 0.3 | 22.9 | 4.4 |
| 60.2 | 140.5 | 136.9 | 26.7 | 72.3 | 13.1 | 3.6 | 12.3 | 1.7 | 10.4 | 1.9 | 5.2 | 0.8 | 4.4 | 0.6 | 39.2 | 5.24 |
| 60.3 | 139.7 | 151.9 | 30.3 | 81.8 | 16.2 | 3.9 | 13.6 | 2 | 13.1 | 2.1 | 4.9 | 0.8 | 4.5 | 0.6 | 44.2 | 5.14 |
| 61 | 100.7 | 93.3 | 19.1 | 47.7 | 10.5 | 1.2 | 5.8 | 0.9 | 5.5 | 0.9 | 3 | 0.6 | 3.5 | 0.3 | 23 | 4.85 |
| 62 | 36.58 | 39.9 | 7.1 | 24.5 | 4.6 | 0.9 | 2.9 | 0.5 | 3.1 | 0.5 | 1.5 | 0.2 | 1.2 | 0.2 | 9.8 | 4.53 |
| 63 | 181.1 | 175.2 | 39 | 106.8 | 22.6 | 5.7 | 14.6 | 2.6 | 15 | 2.4 | 6.8 | 0.9 | 5.8 | 0.7 | 50.6 | 5.49 |
| 63.1 | 152.4 | 175 | 34.7 | 102.5 | 21.4 | 6 | 15.8 | 2.4 | 15.5 | 2.8 | 7 | 1.5 | 7.3 | 0.9 | 58.8 | 5.76 |
| 64 | 97.78 | 113.2 | 23.8 | 72.1 | 16.3 | 3.5 | 12.7 | 1.7 | 10.2 | 1.6 | 4.3 | 0.6 | 3.5 | 0.4 | 37.9 | 5.57 |
| 65 | 36.97 | 44.8 | 5.3 | 16.4 | 2.9 | 0.8 | 3.1 | 0.5 | 3.9 | 0.5 | 1.7 | 0.4 | 2.7 | 0.2 | 14.2 | 4.84 |
| 66 | 74.9 | 93.1 | 15.2 | 39.8 | 7.5 | 1 | 7 | 1 | 6.3 | 1 | 3.2 | 0.6 | 4.1 | 0.6 | 24.4 | 4.49 |
| 67 | 42.98 | 37.8 | 6.6 | 20 | 3.6 | 0.9 | 2.8 | 0.5 | 2.3 | 0.4 | 1.3 | 0.2 | 1.2 | 0.2 | 10.9 | 4.94 |
| 67.1 | 128 | 129 | 20 | 55.3 | 9.1 | 2.2 | 7.2 | 1.1 | 6.6 | 1.1 | 3.2 | 0.5 | 2.8 | 0.2 | 27.4 | 5.34 |
| 67.2 | 88.24 | 94.4 | 12.3 | 31.6 | 5.6 | 1.3 | 4.3 | 0.6 | 4.2 | 0.7 | 2.2 | 0.4 | 2.1 | 0.2 | 15.4 | 4.57 |
| 68 | 84.89 | 99 | 18.9 | 53.4 | 10.8 | 3.5 | 7.9 | 1.2 | 7.4 | 1.3 | 3.4 | 0.4 | 4.5 | 0.3 | 29.6 | 5.34 |
| 69 | 93.16 | 95.4 | 17.4 | 47 | 11.4 | 3.3 | 6.6 | 1.1 | 6.5 | 1.4 | 2.9 | 0.5 | 3.6 | 0.4 | 29 | 4.65 |
| 70 | 28.66 | 29.1 | 7.5 | 23.3 | 5.6 | 1.5 | 5.6 | 1.2 | 7.9 | 1.6 | 4 | 0.7 | 4.1 | 0.7 | 37.8 | 5.44 |

**Table S2**

Calculated Enrichment Factor (EF) for REEs in topsoil samples

|  | ***(EF)*** | | | | | | | | | | | | | |
| --- | --- | --- | --- | --- | --- | --- | --- | --- | --- | --- | --- | --- | --- | --- |
| ***Sample***  ***ID*** | ***La*** | ***Ce*** | ***Pr*** | ***Nd*** | ***Sm*** | ***Eu*** | ***Gd*** | ***Tb*** | ***Dy*** | ***Ho*** | ***Er*** | ***Tm*** | ***Yb*** | ***Lu*** |
| 1 | 0.59 | 0.37 | 0.57 | 0.54 | 0.73 | 0.88 | 0.88 | 1.78 | 1.13 | 1.08 | 1.10 | 1.39 | 0.82 | 0.03 |
| 2 | 1.38 | 1.43 | 1.32 | 1.13 | 1.36 | 1.48 | 1.62 | 0.69 | 1.43 | 1.32 | 1.38 | 1.43 | 1.24 | 0.06 |
| 3 | 3.77 | 2.40 | 2.17 | 1.88 | 1.55 | 1.91 | 1.64 | 0.83 | 1.66 | 1.10 | 1.32 | 1.20 | 1.07 | 0.03 |
| 4 | 2.19 | 1.74 | 1.86 | 1.77 | 2.32 | 2.08 | 2.10 | 0.62 | 1.99 | 1.46 | 1.58 | 1.19 | 1.08 | 0.04 |
| 5 | 2.67 | 2.10 | 2.02 | 1.56 | 1.88 | 1.42 | 2.04 | 0.33 | 1.70 | 1.24 | 1.18 | 1.52 | 1.96 | 0.05 |
| 6 | 2.23 | 1.81 | 2.05 | 1.96 | 1.80 | 2.80 | 2.24 | 0.96 | 1.36 | 1.47 | 1.06 | 1.03 | 1.04 | 0.04 |
| 7 | 0.57 | 1.49 | 1.55 | 1.28 | 1.70 | 2.12 | 1.79 | 1.36 | 1.47 | 1.19 | 1.19 | 1.46 | 1.07 | 0.02 |
| 8 | 1.42 | 1.69 | 1.86 | 1.57 | 1.80 | 2.31 | 1.70 | 0.68 | 1.25 | 1.18 | 1.24 | 2.17 | 1.40 | 0.04 |
| 9 | 0.96 | 1.00 | 1.17 | 1.00 | 1.81 | 1.48 | 1.52 | 0.87 | 1.32 | 1.04 | 1.61 | 1.69 | 1.42 | 0.04 |
| 10 | 1.38 | 1.60 | 1.73 | 1.35 | 2.01 | 1.82 | 1.75 | 0.66 | 1.71 | 1.07 | 1.38 | 1.49 | 1.44 | 0.04 |
| 11 | 0.62 | 1.04 | 1.16 | 0.99 | 1.33 | 1.37 | 1.27 | 1.01 | 1.81 | 1.09 | 1.66 | 1.46 | 2.06 | 0.04 |
| 12 | 1.67 | 1.35 | 1.59 | 1.59 | 1.78 | 1.66 | 1.90 | 0.89 | 1.34 | 1.02 | 1.49 | 1.38 | 1.23 | 0.04 |
| 13 | 1.97 | 1.50 | 1.65 | 1.51 | 1.93 | 1.80 | 1.57 | 0.41 | 1.35 | 1.23 | 1.11 | 1.20 | 1.47 | 0.04 |
| 14 | 0.90 | 1.52 | 1.90 | 1.75 | 2.16 | 1.81 | 1.71 | 0.67 | 1.31 | 1.36 | 1.16 | 1.48 | 1.01 | 0.05 |
| 15 | 0.63 | 1.31 | 1.50 | 1.60 | 1.68 | 1.90 | 1.80 | 1.07 | 1.68 | 1.60 | 1.44 | 1.61 | 1.16 | 0.03 |
| 16 | 0.77 | 1.33 | 1.53 | 1.58 | 1.76 | 2.19 | 2.01 | 0.93 | 1.70 | 1.09 | 1.28 | 1.33 | 1.11 | 0.05 |
| 17 | 0.77 | 1.28 | 1.44 | 1.44 | 1.71 | 2.32 | 1.54 | 0.96 | 1.31 | 1.26 | 1.32 | 1.03 | 0.89 | 0.04 |
| 18 | 1.53 | 1.32 | 1.45 | 1.31 | 1.74 | 2.09 | 1.22 | 0.93 | 1.61 | 1.19 | 1.13 | 1.33 | 1.37 | 0.03 |
| 19 | 1.26 | 0.89 | 0.92 | 0.93 | 1.44 | 1.37 | 1.40 | 0.81 | 1.24 | 1.24 | 1.08 | 1.52 | 1.09 | 0.04 |
| 20 | 1.30 | 1.21 | 1.00 | 1.06 | 1.35 | 1.64 | 1.67 | 1.01 | 1.68 | 1.49 | 1.35 | 1.70 | 1.43 | 0.04 |
| 21 | 2.70 | 1.47 | 1.27 | 1.08 | 1.16 | 1.68 | 1.48 | 0.61 | 1.44 | 1.50 | 2.02 | 2.03 | 1.52 | 0.06 |
| 22 | 2.12 | 1.76 | 1.19 | 1.10 | 2.01 | 1.53 | 1.15 | 0.49 | 1.98 | 1.46 | 1.59 | 2.54 | 2.19 | 0.05 |
| 23 | 1.07 | 1.75 | 1.68 | 1.66 | 1.86 | 1.65 | 1.70 | 0.62 | 1.55 | 1.47 | 1.02 | 1.60 | 1.38 | 0.07 |
| 24 | 0.44 | 1.03 | 0.96 | 1.07 | 1.35 | 1.38 | 1.35 | 1.14 | 1.40 | 1.06 | 1.07 | 1.08 | 1.24 | 0.04 |
| 25 | 0.51 | 1.59 | 1.44 | 1.50 | 1.51 | 1.63 | 1.47 | 0.97 | 1.59 | 1.46 | 1.34 | 1.28 | 1.32 | 0.04 |
| 26 | 0.31 | 2.35 | 1.93 | 1.64 | 1.39 | 2.25 | 1.50 | 0.70 | 1.53 | 1.15 | 1.25 | 1.06 | 1.37 | 0.04 |
| 27 | 0.40 | 4.38 | 3.11 | 2.29 | 2.47 | 2.65 | 2.27 | 0.66 | 1.58 | 1.07 | 1.32 | 0.74 | 1.44 | 0.05 |
| 28 | 2.59 | 5.79 | 4.12 | 2.98 | 2.19 | 2.54 | 2.81 | 0.51 | 1.76 | 1.18 | 1.24 | 1.45 | 0.97 | 0.05 |
| 30 | 2.40 | 2.58 | 3.06 | 3.04 | 2.98 | 3.44 | 2.39 | 0.30 | 1.73 | 1.36 | 1.66 | 2.50 | 1.91 | 0.12 |
| 31 | 2.59 | 3.15 | 4.14 | 3.19 | 4.32 | 2.78 | 2.63 | 0.37 | 2.04 | 1.10 | 1.72 | 2.02 | 2.42 | 0.07 |
| 32 | 1.13 | 1.09 | 2.07 | 1.50 | 2.10 | 1.72 | 1.90 | 0.64 | 1.66 | 1.10 | 1.14 | 1.15 | 1.21 | 0.04 |
| 33 | 1.42 | 2.04 | 3.30 | 2.86 | 3.95 | 3.35 | 2.59 | 0.41 | 2.06 | 0.97 | 1.10 | 1.79 | 1.97 | 0.06 |
| 34 | 2.07 | 2.23 | 3.31 | 2.25 | 3.14 | 2.95 | 2.00 | 0.41 | 1.89 | 1.24 | 1.29 | 1.82 | 1.13 | 0.04 |
| 35 | 2.09 | 1.72 | 2.93 | 1.74 | 2.15 | 2.37 | 2.13 | 0.35 | 1.82 | 1.15 | 1.40 | 2.11 | 1.51 | 0.02 |
| 36 | 0.94 | 1.54 | 2.13 | 1.45 | 2.10 | 2.00 | 2.27 | 0.46 | 1.38 | 1.31 | 1.44 | 2.13 | 1.53 | 0.04 |
| 37 | 1.13 | 1.56 | 1.94 | 1.40 | 1.90 | 2.07 | 1.58 | 0.76 | 0.70 | 0.40 | 0.60 | 0.98 | 1.54 | 0.05 |
| 38 | 2.54 | 2.11 | 1.86 | 1.09 | 0.81 | 1.47 | 1.65 | 0.38 | 1.07 | 0.80 | 0.93 | 1.31 | 1.22 | 0.05 |
| 39 | 1.87 | 2.22 | 3.28 | 1.80 | 2.07 | 2.21 | 2.22 | 0.25 | 1.61 | 1.20 | 1.39 | 1.96 | 1.83 | 0.07 |
| 40 | 2.10 | 2.49 | 2.67 | 1.75 | 1.40 | 1.60 | 2.13 | 0.46 | 1.80 | 1.09 | 1.44 | 1.60 | 1.30 | 0.06 |
| 41 | 4.47 | 2.21 | 2.81 | 2.11 | 2.10 | 1.69 | 1.96 | 0.27 | 1.95 | 1.47 | 1.54 | 1.80 | 1.81 | 0.03 |
| 42 | 1.48 | 1.88 | 1.96 | 1.40 | 1.69 | 2.00 | 1.85 | 0.28 | 1.46 | 1.09 | 1.64 | 0.89 | 1.91 | 0.06 |
| 43 | 1.06 | 1.60 | 2.61 | 1.55 | 1.99 | 2.70 | 1.91 | 0.65 | 1.80 | 1.08 | 1.08 | 1.14 | 1.36 | 0.03 |
| 44 | 2.65 | 1.46 | 1.86 | 1.49 | 1.57 | 2.22 | 1.47 | 0.59 | 1.75 | 1.21 | 0.84 | 1.27 | 1.03 | 0.04 |
| 45 | 2.32 | 1.30 | 1.67 | 1.12 | 1.40 | 1.89 | 1.32 | 0.24 | 1.40 | 1.65 | 1.00 | 2.02 | 1.31 | 0.03 |
| 46 | 0.95 | 1.09 | 1.60 | 1.19 | 1.69 | 1.78 | 1.11 | 0.83 | 1.59 | 1.09 | 0.93 | 1.48 | 1.11 | 0.03 |
| 47 | 0.69 | 1.04 | 1.71 | 1.21 | 1.36 | 2.33 | 1.17 | 0.71 | 1.46 | 0.99 | 0.98 | 1.38 | 1.34 | 0.04 |
| 48 | 0.90 | 0.90 | 1.68 | 1.00 | 1.39 | 2.55 | 1.49 | 1.13 | 1.58 | 1.08 | 1.00 | 1.54 | 1.04 | 0.03 |
| 49 | 0.67 | 0.85 | 1.39 | 1.13 | 1.77 | 1.56 | 1.96 | 0.71 | 1.62 | 1.56 | 1.13 | 2.08 | 1.40 | 0.04 |
| 50 | 0.39 | 0.98 | 1.96 | 1.35 | 2.04 | 2.77 | 1.61 | 1.10 | 1.51 | 1.28 | 1.02 | 1.12 | 1.25 | 0.04 |
| 52 | 1.03 | 1.08 | 2.03 | 1.62 | 1.95 | 2.79 | 1.86 | 1.06 | 1.63 | 1.14 | 1.32 | 1.63 | 1.60 | 0.03 |
| 53 | 1.16 | 1.05 | 1.72 | 1.40 | 1.66 | 2.56 | 1.44 | 0.43 | 2.02 | 1.16 | 1.21 | 1.14 | 1.39 | 0.04 |
| 56 | 2.12 | 0.97 | 1.77 | 1.51 | 1.51 | 2.38 | 1.88 | 1.24 | 1.56 | 1.05 | 0.99 | 1.19 | 1.14 | 0.03 |
| 59 | 2.23 | 1.12 | 1.95 | 1.38 | 1.95 | 2.27 | 1.05 | 0.20 | 1.47 | 1.49 | 1.21 | 1.21 | 0.87 | 0.04 |
| 60 | 0.48 | 0.96 | 1.76 | 1.17 | 1.57 | 1.78 | 1.24 | 1.14 | 1.42 | 1.41 | 1.26 | 1.29 | 1.39 | 0.03 |
| 60.1 | 0.32 | 1.16 | 2.26 | 1.72 | 2.12 | 2.29 | 1.47 | 0.85 | 1.71 | 1.55 | 1.28 | 1.16 | 1.13 | 0.03 |
| 60.2 | 0.19 | 1.18 | 2.08 | 1.47 | 1.63 | 2.30 | 1.82 | 1.45 | 1.53 | 1.32 | 1.26 | 1.36 | 1.07 | 0.04 |
| 60.3 | 0.23 | 1.17 | 2.10 | 1.47 | 1.78 | 2.21 | 1.78 | 1.64 | 1.71 | 1.29 | 1.05 | 1.21 | 0.97 | 0.03 |
| 61 | 1.23 | 1.38 | 2.54 | 1.65 | 2.22 | 1.30 | 1.46 | 0.85 | 1.38 | 1.07 | 1.24 | 1.74 | 1.46 | 0.03 |
| 62 | 0.58 | 1.38 | 2.22 | 1.99 | 2.28 | 2.30 | 1.71 | 0.36 | 1.82 | 1.39 | 1.45 | 1.36 | 1.17 | 0.05 |
| 63 | 0.13 | 1.17 | 2.36 | 1.68 | 2.17 | 2.82 | 1.67 | 1.87 | 1.71 | 1.29 | 1.27 | 1.19 | 1.10 | 0.03 |
| 63.1 | 0.18 | 1.01 | 1.80 | 1.39 | 1.77 | 2.55 | 1.56 | 2.18 | 1.52 | 1.30 | 1.13 | 1.70 | 1.19 | 0.04 |
| 64 | 0.74 | 1.01 | 1.92 | 1.52 | 2.09 | 2.31 | 1.94 | 1.40 | 1.55 | 1.15 | 1.07 | 1.06 | 0.88 | 0.02 |
| 65 | 0.97 | 1.07 | 1.14 | 0.92 | 0.99 | 1.41 | 1.26 | 0.53 | 1.58 | 0.96 | 1.13 | 1.88 | 1.82 | 0.03 |
| 66 | 0.98 | 1.29 | 1.90 | 1.30 | 1.50 | 1.02 | 1.66 | 0.90 | 1.49 | 1.12 | 1.24 | 1.64 | 1.61 | 0.06 |
| 67 | 0.74 | 1.18 | 1.85 | 1.46 | 1.61 | 2.06 | 1.49 | 0.40 | 1.22 | 1.00 | 1.13 | 1.22 | 1.05 | 0.04 |
| 67.1 | 0.43 | 1.60 | 2.23 | 1.61 | 1.62 | 2.01 | 1.52 | 1.01 | 1.39 | 1.09 | 1.11 | 1.22 | 0.98 | 0.02 |
| 67.2 | 0.79 | 2.08 | 2.44 | 1.63 | 1.77 | 2.11 | 1.62 | 0.57 | 1.57 | 1.24 | 1.35 | 1.73 | 1.31 | 0.03 |
| 68 | 0.37 | 1.13 | 1.95 | 1.44 | 1.78 | 2.96 | 1.55 | 1.10 | 1.44 | 1.20 | 1.09 | 0.90 | 1.46 | 0.02 |
| 69 | 1.24 | 1.12 | 1.83 | 1.29 | 1.91 | 2.84 | 1.32 | 1.07 | 1.29 | 1.32 | 0.95 | 1.15 | 1.19 | 0.03 |
| 70 | 0.54 | 0.26 | 0.61 | 0.49 | 0.72 | 0.99 | 0.86 | 1.40 | 1.21 | 1.15 | 1.00 | 1.23 | 1.04 | 0.04 |

**Table S3**

Calculated Geoacummulation index (Igeo) for REEs in topsoil samples

|  | ***(Igeo)*** | | | | | | | | | | | | | |  |
| --- | --- | --- | --- | --- | --- | --- | --- | --- | --- | --- | --- | --- | --- | --- | --- |
| ***Sample***  ***ID*** | ***La*** | ***Ce*** | ***Pr*** | ***Nd*** | ***Sm*** | ***Eu*** | ***Gd*** | ***Tb*** | ***Dy*** | ***Ho*** | ***Er*** | ***Tm*** | ***Yb*** | ***Lu*** | ***Y*** |
| 1 | -1.43 | -1.44 | -1.29 | -1.09 | -1.01 | -1.35 | -0.74 | -1.02 | -0.44 | -0.31 | -0.24 | -0.10 | -0.53 | -1.28 | -0.13 |
| 2 | -1.53 | -1.02 | -1.41 | -1.31 | -1.33 | -1.13 | -1.07 | -1.34 | -1.16 | -1.05 | -0.97 | -1.02 | -1.07 | -1.62 | -1.08 |
| 3 | -0.34 | -0.32 | -0.73 | -0.62 | -1.02 | -1.27 | -0.88 | -1.11 | -0.83 | -1.05 | -0.83 | -1.02 | -1.03 | -2.13 | -0.90 |
| 4 | -1.17 | -0.93 | -1.17 | -0.96 | -0.90 | -1.64 | -0.91 | -1.34 | -0.93 | -1.05 | -0.93 | -1.31 | -1.30 | -2.13 | -1.19 |
| 5 | -1.62 | -1.39 | -1.73 | -1.74 | -1.76 | -1.54 | -1.59 | -2.03 | -1.73 | -1.86 | -1.86 | -1.71 | -1.36 | -2.53 | -1.83 |
| 6 | -0.72 | -0.46 | -0.64 | -0.43 | -0.72 | -1.01 | -0.42 | -0.85 | -0.88 | -0.61 | -0.89 | -1.02 | -0.91 | -1.84 | -0.75 |
| 7 | -0.46 | -0.30 | -0.57 | -0.51 | -0.43 | -2.05 | -0.30 | -0.64 | -0.45 | -0.48 | -0.43 | -0.33 | -0.53 | -2.13 | -0.41 |
| 8 | -1.21 | -0.87 | -1.08 | -0.99 | -1.06 | -2.05 | -1.04 | -1.11 | -1.30 | -1.17 | -1.08 | -0.62 | -0.95 | -2.13 | -1.09 |
| 9 | -1.44 | -1.14 | -1.29 | -1.19 | -0.81 | -1.76 | -0.90 | -1.11 | -1.00 | -1.05 | -0.57 | -0.62 | -0.69 | -1.84 | -0.85 |
| 10 | -1.30 | -0.95 | -1.18 | -1.17 | -0.98 | -2.23 | -1.04 | -1.34 | -1.02 | -1.30 | -1.00 | -1.02 | -0.95 | -2.13 | -1.12 |
| 11 | -1.39 | -0.96 | -1.15 | -1.05 | -0.97 | -2.05 | -0.93 | -0.85 | -0.53 | -0.85 | -0.39 | -0.62 | -0.17 | -1.62 | -0.70 |
| 12 | -1.01 | -0.82 | -0.97 | -0.71 | -0.81 | -0.90 | -0.66 | -0.93 | -0.96 | -1.05 | -0.62 | -0.80 | -0.81 | -1.84 | -0.82 |
| 13 | -1.88 | -1.49 | -1.70 | -1.53 | -1.50 | -1.20 | -1.62 | -1.62 | -1.73 | -1.64 | -1.70 | -1.71 | -1.41 | -2.53 | -1.60 |
| 14 | -1.27 | -1.00 | -1.08 | -0.91 | -0.90 | -2.23 | -1.06 | -1.22 | -1.27 | -1.05 | -1.17 | -1.02 | -1.30 | -1.84 | -1.12 |
| 15 | -0.97 | -0.67 | -0.84 | -0.52 | -0.68 | -1.07 | -0.53 | -0.85 | -0.56 | -0.42 | -0.48 | -0.46 | -0.69 | -1.84 | -0.64 |
| 16 | -1.08 | -0.80 | -0.96 | -0.67 | -0.77 | -0.95 | -0.56 | -0.53 | -0.68 | -0.95 | -0.74 | -0.80 | -0.88 | -1.62 | -0.78 |
| 17 | -1.15 | -0.81 | -0.99 | -0.73 | -0.77 | -0.41 | -0.79 | -0.93 | -0.91 | -0.77 | -0.68 | -1.02 | -1.07 | -1.84 | -0.75 |
| 18 | -1.17 | -0.80 | -1.02 | -0.86 | -0.78 | -1.35 | -1.06 | -1.02 | -0.74 | -0.85 | -0.86 | -0.80 | -0.66 | -2.13 | -0.78 |
| 19 | -1.83 | -1.33 | -1.60 | -1.34 | -1.11 | -0.34 | -1.06 | -1.47 | -1.14 | -0.95 | -1.04 | -0.80 | -1.03 | -1.84 | -0.92 |
| 20 | -1.50 | -0.80 | -1.29 | -0.98 | -0.95 | -0.37 | -0.66 | -0.93 | -0.61 | -0.54 | -0.60 | -0.46 | -0.53 | -1.62 | -0.70 |
| 21 | -1.75 | -1.12 | -1.58 | -1.48 | -1.62 | -1.35 | -1.29 | -1.47 | -1.27 | -1.05 | -0.71 | -0.80 | -0.99 | -1.84 | -1.21 |
| 22 | -1.97 | -1.17 | -1.86 | -1.69 | -1.29 | -0.37 | -1.77 | -1.62 | -1.18 | -1.30 | -1.17 | -0.80 | -0.85 | -2.13 | -1.43 |
| 23 | -1.50 | -0.93 | -1.27 | -1.03 | -1.12 | -2.23 | -1.13 | -1.11 | -1.18 | -1.05 | -1.37 | -1.02 | -1.07 | -1.62 | -1.19 |
| 24 | -1.07 | -0.84 | -1.22 | -0.86 | -0.83 | -0.75 | -0.75 | -0.71 | -0.67 | -0.77 | -0.71 | -0.80 | -0.56 | -1.44 | -0.58 |
| 25 | -0.80 | -0.58 | -0.98 | -0.68 | -0.89 | -0.79 | -0.84 | -0.93 | -0.71 | -0.61 | -0.65 | -0.80 | -0.66 | -1.84 | -0.74 |
| 26 | -0.77 | -0.51 | -1.02 | -0.92 | -1.29 | -0.26 | -1.13 | -1.47 | -1.08 | -1.17 | -1.04 | -1.31 | -0.95 | -2.13 | -1.07 |
| 27 | 0.06 | 0.06 | -0.59 | -0.64 | -0.77 | -0.18 | -0.78 | -1.02 | -1.10 | -1.30 | -1.04 | -1.71 | -0.95 | -1.84 | -1.12 |
| 28 | -0.16 | 0.08 | -0.57 | -0.64 | -1.16 | -1.35 | -0.82 | -1.34 | -1.25 | -1.46 | -1.37 | -1.31 | -1.61 | -2.13 | -1.38 |
| 30 | -1.76 | -1.28 | -1.41 | -1.17 | -1.39 | -1.64 | -1.53 | -1.62 | -1.81 | -1.86 | -1.62 | -1.31 | -1.47 | -1.84 | -1.93 |
| 31 | -1.13 | -0.86 | -0.90 | -0.90 | -0.81 | 0.20 | -1.22 | -1.34 | -1.43 | -1.86 | -1.37 | -1.31 | -1.03 | -2.13 | -1.71 |
| 32 | -1.43 | -1.36 | -1.02 | -1.10 | -0.97 | 0.25 | -0.98 | -1.47 | -1.08 | -1.30 | -1.22 | -1.31 | -1.16 | -2.13 | -1.15 |
| 33 | -1.16 | -1.17 | -1.00 | -0.89 | -0.77 | -0.28 | -1.11 | -1.34 | -1.30 | -1.86 | -1.70 | -1.31 | -1.11 | -2.13 | -1.59 |
| 34 | -0.95 | -1.10 | -1.02 | -1.15 | -1.02 | -1.76 | -1.39 | -1.62 | -1.40 | -1.64 | -1.55 | -1.31 | -1.68 | -2.53 | -1.61 |
| 35 | -1.31 | -1.51 | -1.28 | -1.55 | -1.55 | -1.54 | -1.47 | -1.81 | -1.59 | -1.86 | -1.62 | -1.31 | -1.54 | -3.23 | -1.76 |
| 36 | -1.17 | -1.35 | -1.33 | -1.46 | -1.29 | -1.64 | -1.13 | -1.34 | -1.59 | -1.46 | -1.32 | -1.02 | -1.25 | -2.53 | -1.48 |
| 37 | -0.64 | -0.84 | -0.93 | -1.00 | -0.90 | -0.75 | -1.00 | -1.34 | -1.77 | -2.15 | -1.70 | -1.31 | -0.75 | -1.84 | -0.99 |
| 38 | -1.33 | -1.24 | -1.67 | -1.94 | -2.45 | -1.27 | -1.66 | -2.32 | -2.04 | -2.15 | -1.96 | -1.71 | -1.68 | -2.53 | -1.68 |
| 39 | -1.44 | -1.59 | -1.50 | -1.85 | -1.92 | -0.28 | -1.77 | -2.72 | -2.04 | -2.15 | -1.96 | -1.71 | -1.68 | -2.53 | -2.09 |
| 40 | -0.72 | -0.87 | -1.10 | -1.27 | -1.70 | -0.34 | -1.20 | -2.32 | -1.32 | -1.64 | -1.32 | -1.31 | -1.41 | -2.13 | -1.48 |
| 41 | -1.45 | -1.51 | -1.58 | -1.61 | -1.82 | -1.13 | -1.81 | -2.32 | -1.77 | -1.86 | -1.78 | -1.71 | -1.61 | -3.23 | -2.01 |
| 42 | -1.68 | -1.66 | -1.92 | -2.00 | -2.03 | -2.05 | -1.85 | -2.03 | -2.04 | -2.15 | -1.70 | -2.41 | -1.54 | -2.53 | -1.99 |
| 43 | -0.59 | -0.96 | -0.78 | -1.05 | -1.01 | -0.90 | -0.97 | -1.11 | -0.98 | -1.30 | -1.27 | -1.31 | -1.03 | -2.53 | -1.14 |
| 44 | -1.10 | -1.17 | -1.23 | -1.20 | -1.35 | -1.20 | -1.34 | -1.34 | -1.12 | -1.30 | -1.62 | -1.31 | -1.41 | -2.13 | -1.25 |
| 45 | -1.92 | -2.15 | -2.21 | -2.35 | -2.34 | -2.23 | -2.32 | -2.32 | -2.21 | -1.86 | -2.32 | -1.71 | -2.05 | -3.23 | -2.12 |
| 46 | -0.91 | -1.10 | -1.02 | -1.07 | -0.93 | -1.07 | -1.26 | -1.34 | -0.86 | -1.05 | -1.17 | -0.80 | -0.99 | -2.13 | -0.89 |
| 47 | -1.24 | -1.30 | -1.11 | -1.21 | -1.29 | -0.95 | -1.36 | -1.34 | -1.10 | -1.30 | -1.27 | -1.02 | -0.95 | -2.13 | -1.05 |
| 48 | -0.77 | -0.99 | -0.68 | -0.94 | -0.82 | -0.41 | -0.67 | -1.02 | -0.57 | -0.77 | -0.80 | -0.46 | -0.75 | -1.84 | -0.59 |
| 49 | -1.50 | -1.51 | -1.33 | -1.28 | -1.04 | -1.35 | -0.85 | -0.64 | -1.00 | -0.85 | -1.13 | -0.62 | -0.91 | -2.13 | -1.05 |
| 50 | -0.74 | -0.93 | -0.54 | -0.66 | -0.46 | -0.34 | -0.61 | -0.64 | -0.63 | -0.61 | -0.80 | -0.80 | -0.58 | -1.62 | -0.61 |
| 52 | -0.62 | -0.87 | -0.54 | -0.52 | -0.54 | -0.37 | -0.50 | -0.85 | -0.59 | -0.77 | -0.57 | -0.46 | -0.38 | -1.84 | -0.65 |
| 53 | -1.57 | -1.80 | -1.60 | -1.56 | -1.59 | -1.35 | -1.66 | -2.03 | -1.27 | -1.64 | -1.55 | -1.71 | -1.41 | -2.53 | -1.55 |
| 56 | -0.79 | -0.83 | -0.52 | -0.43 | -0.64 | -0.37 | -0.33 | -0.64 | -0.48 | -0.69 | -0.71 | -0.62 | -0.56 | -1.84 | -0.49 |
| 59 | -2.45 | -2.49 | -2.24 | -2.33 | -2.19 | -2.23 | -2.72 | -2.72 | -2.35 | -2.15 | -2.32 | -2.41 | -2.64 | -3.23 | -2.30 |
| 60 | -0.69 | -0.92 | -0.61 | -0.77 | -0.68 | -0.75 | -0.84 | -1.11 | -0.66 | -0.48 | -0.55 | -0.62 | -0.44 | -1.84 | -0.58 |
| 60.1 | -0.88 | -1.03 | -0.66 | -0.68 | -0.68 | -0.79 | -0.97 | -0.93 | -0.77 | -0.69 | -0.83 | -1.02 | -0.95 | -2.13 | -0.88 |
| 60.2 | -0.17 | -0.47 | -0.21 | -0.30 | -0.41 | -0.26 | -0.21 | -0.58 | -0.34 | -0.31 | -0.31 | -0.33 | -0.46 | -1.44 | -0.34 |
| 60.3 | -0.18 | -0.36 | -0.08 | -0.18 | -0.20 | -0.18 | -0.11 | -0.42 | -0.11 | -0.21 | -0.37 | -0.33 | -0.44 | -1.44 | -0.22 |
| 61 | -0.51 | -0.85 | -0.54 | -0.72 | -0.63 | -1.35 | -0.97 | -1.22 | -0.98 | -1.05 | -0.86 | -0.62 | -0.69 | -2.13 | -0.87 |
| 62 | -1.52 | -1.70 | -1.53 | -1.38 | -1.46 | -1.64 | -1.66 | -1.81 | -1.55 | -1.64 | -1.55 | -1.71 | -1.76 | -2.53 | -1.72 |
| 63 | 0.08 | -0.22 | 0.17 | 0.09 | 0.14 | 0.20 | -0.04 | -0.16 | 0.02 | -0.07 | -0.04 | -0.21 | -0.19 | -1.28 | -0.08 |
| 63.1 | -0.09 | -0.22 | 0.05 | 0.05 | 0.08 | 0.25 | 0.04 | -0.24 | 0.06 | 0.08 | -0.01 | 0.30 | 0.04 | -1.03 | 0.07 |
| 64 | -0.54 | -0.66 | -0.32 | -0.30 | -0.19 | -0.28 | -0.18 | -0.58 | -0.36 | -0.48 | -0.50 | -0.62 | -0.69 | -1.84 | -0.37 |
| 65 | -1.51 | -1.58 | -1.82 | -1.79 | -1.92 | -1.76 | -1.59 | -1.81 | -1.32 | -1.64 | -1.43 | -1.02 | -0.95 | -2.53 | -1.35 |
| 66 | -0.80 | -0.85 | -0.77 | -0.90 | -0.97 | -1.54 | -0.78 | -1.11 | -0.84 | -0.95 | -0.80 | -0.62 | -0.53 | -1.44 | -0.81 |
| 67 | -1.36 | -1.75 | -1.60 | -1.59 | -1.70 | -1.64 | -1.69 | -1.81 | -1.85 | -1.86 | -1.70 | -1.71 | -1.76 | -2.53 | -1.62 |
| 67.1 | -0.27 | -0.53 | -0.50 | -0.57 | -0.77 | -0.75 | -0.75 | -1.02 | -0.80 | -0.85 | -0.80 | -0.80 | -0.91 | -2.53 | -0.70 |
| 67.2 | -0.64 | -0.84 | -0.98 | -1.13 | -1.26 | -1.27 | -1.26 | -1.62 | -1.25 | -1.30 | -1.17 | -1.02 | -1.20 | -2.53 | -1.27 |
| 68 | -0.68 | -0.79 | -0.55 | -0.60 | -0.60 | -0.28 | -0.66 | -0.93 | -0.68 | -0.69 | -0.74 | -1.02 | -0.44 | -2.13 | -0.62 |
| 69 | -0.59 | -0.83 | -0.64 | -0.73 | -0.55 | -0.34 | -0.84 | -1.02 | -0.81 | -0.61 | -0.89 | -0.80 | -0.66 | -1.84 | -0.64 |
| 70 | -1.76 | -2.01 | -1.48 | -1.43 | -1.26 | -1.13 | -1.00 | -0.93 | -0.62 | -0.48 | -0.57 | -0.46 | -0.53 | -1.28 | -0.37 |

**Table S4**

REEs characteristics of topsoils from Ditrău Alkaline Massif area

| ***Sample***  ***ID*** | ***LREE/HREE*** | ***δCe **** | ***δEu **** | ***δGd*** | ***δTb*** | ***δHo*** | ***(La/Yb)_ch_*** | ***(La/Sm)_ch_*** | ***(Gd/Yb)_ch_*** |
| --- | --- | --- | --- | --- | --- | --- | --- | --- | --- |
| 1 | 4.59 | 0.64 | 0.71 | 1.20 | 0.82 | 0.95 | 7.00 | 3.59 | 1.47 |
| 2 | 8.88 | 1.08 | 0.64 | 1.25 | 1.00 | 0.92 | 10.85 | 4.51 | 1.79 |
| 3 | 16.95 | 0.77 | 0.76 | 1.09 | 0.97 | 0.72 | 34.14 | 10.82 | 2.08 |
| 4 | 10.32 | 0.86 | 0.59 | 1.30 | 0.82 | 0.80 | 19.67 | 4.20 | 2.66 |
| 5 | 11.68 | 0.88 | 0.46 | 1.55 | 0.85 | 0.84 | 13.23 | 6.30 | 1.42 |
| 6 | 12.39 | 0.85 | 0.90 | 1.14 | 0.97 | 1.19 | 20.93 | 5.49 | 2.95 |
| 7 | 10.71 | 0.82 | 0.77 | 1.14 | 0.94 | 0.88 | 18.47 | 5.33 | 2.28 |
| 8 | 11.47 | 0.91 | 0.83 | 0.93 | 1.29 | 0.93 | 13.20 | 4.72 | 1.65 |
| 9 | 7.19 | 0.87 | 0.55 | 1.18 | 1.05 | 0.70 | 8.12 | 2.91 | 1.47 |
| 10 | 9.84 | 0.92 | 0.60 | 1.19 | 0.92 | 0.68 | 12.11 | 3.98 | 1.65 |
| 11 | 6.22 | 0.96 | 0.66 | 0.96 | 1.11 | 0.62 | 5.08 | 3.60 | 0.84 |
| 12 | 9.72 | 0.81 | 0.57 | 1.28 | 1.07 | 0.71 | 14.07 | 4.46 | 2.11 |
| 13 | 10.60 | 0.94 | 0.64 | 0.97 | 1.31 | 0.98 | 10.80 | 3.76 | 1.46 |
| 14 | 11.65 | 0.83 | 0.57 | 1.10 | 1.16 | 1.08 | 17.78 | 3.80 | 2.31 |
| 15 | 8.85 | 0.88 | 0.69 | 1.20 | 0.91 | 1.01 | 13.03 | 4.11 | 2.13 |
| 16 | 8.74 | 0.86 | 0.75 | 0.98 | 1.36 | 0.71 | 14.10 | 4.04 | 2.48 |
| 17 | 10.05 | 0.90 | 0.89 | 0.91 | 1.14 | 0.94 | 15.87 | 3.77 | 2.38 |
| 18 | 9.78 | 0.93 | 0.86 | 0.78 | 1.11 | 0.85 | 10.34 | 3.72 | 1.22 |
| 19 | 7.06 | 1.03 | 0.61 | 1.30 | 0.85 | 1.05 | 7.69 | 2.66 | 1.75 |
| 20 | 6.85 | 1.26 | 0.70 | 1.21 | 0.93 | 0.96 | 6.56 | 3.19 | 1.59 |
| 21 | 7.93 | 1.20 | 0.83 | 1.08 | 1.03 | 0.85 | 8.00 | 4.80 | 1.34 |
| 22 | 8.38 | 1.46 | 0.58 | 0.84 | 1.07 | 0.80 | 5.57 | 2.79 | 0.72 |
| 23 | 10.66 | 1.11 | 0.58 | 1.04 | 1.30 | 1.11 | 11.12 | 3.75 | 1.69 |
| 24 | 8.19 | 0.89 | 0.64 | 1.00 | 1.26 | 0.84 | 10.33 | 4.32 | 1.49 |
| 25 | 10.96 | 0.89 | 0.69 | 1.04 | 1.07 | 0.98 | 15.04 | 6.02 | 1.52 |
| 26 | 15.49 | 0.95 | 0.99 | 0.99 | 0.87 | 0.81 | 20.62 | 9.28 | 1.50 |
| 27 | 25.07 | 0.80 | 0.70 | 1.09 | 1.11 | 0.72 | 47.08 | 12.57 | 2.14 |
| 28 | 28.37 | 0.96 | 0.66 | 1.42 | 0.88 | 0.77 | 73.32 | 14.90 | 3.96 |
| 30 | 12.90 | 0.96 | 0.79 | 0.91 | 1.27 | 0.79 | 12.96 | 3.81 | 1.71 |
| 31 | 14.80 | 0.82 | 0.48 | 1.08 | 1.21 | 0.57 | 15.44 | 3.95 | 1.49 |
| 32 | 8.79 | 0.62 | 0.54 | 1.40 | 0.80 | 0.76 | 13.11 | 3.46 | 2.14 |
| 33 | 12.69 | 0.63 | 0.62 | 1.02 | 1.08 | 0.60 | 16.31 | 3.71 | 1.80 |
| 34 | 15.98 | 0.59 | 0.69 | 0.96 | 0.99 | 0.76 | 35.75 | 5.89 | 2.42 |
| 35 | 12.00 | 0.55 | 0.70 | 1.17 | 0.94 | 0.70 | 21.62 | 6.95 | 1.93 |
| 36 | 10.79 | 0.59 | 0.58 | 1.43 | 0.91 | 0.91 | 19.00 | 6.33 | 2.03 |
| 37 | 16.61 | 0.61 | 0.74 | 1.07 | 0.91 | 0.96 | 19.10 | 7.09 | 1.40 |
| 38 | 16.09 | 0.82 | 0.82 | 1.53 | 0.68 | 0.60 | 24.50 | 16.86 | 1.85 |
| 39 | 14.35 | 0.59 | 0.65 | 1.61 | 0.54 | 0.78 | 21.97 | 8.86 | 1.65 |
| 40 | 15.95 | 0.66 | 0.60 | 2.04 | 0.43 | 0.66 | 34.18 | 14.53 | 2.24 |
| 41 | 13.68 | 0.66 | 0.52 | 1.45 | 0.74 | 0.83 | 20.17 | 7.94 | 1.48 |
| 42 | 11.59 | 0.74 | 0.72 | 1.10 | 1.13 | 0.69 | 14.92 | 7.75 | 1.32 |
| 43 | 13.01 | 0.50 | 0.87 | 0.94 | 1.08 | 0.73 | 26.61 | 8.32 | 1.92 |
| 44 | 12.07 | 0.66 | 0.91 | 0.85 | 1.13 | 0.90 | 23.41 | 7.02 | 1.95 |
| 45 | 11.44 | 0.58 | 0.87 | 0.87 | 1.19 | 1.34 | 19.61 | 8.36 | 1.38 |
| 46 | 10.93 | 0.58 | 0.77 | 0.85 | 0.95 | 0.84 | 18.58 | 5.58 | 1.37 |
| 47 | 9.87 | 0.61 | 1.14 | 0.72 | 1.13 | 0.79 | 12.83 | 5.77 | 1.19 |
| 48 | 8.68 | 0.53 | 1.13 | 0.91 | 0.84 | 0.82 | 16.90 | 5.77 | 1.96 |
| 49 | 6.31 | 0.63 | 0.53 | 0.99 | 1.64 | 1.11 | 9.60 | 3.46 | 1.92 |
| 50 | 9.26 | 0.53 | 0.93 | 0.81 | 1.22 | 0.99 | 14.70 | 4.13 | 1.76 |
| 52 | 9.19 | 0.52 | 0.92 | 0.99 | 0.92 | 0.76 | 13.39 | 5.03 | 1.59 |
| 53 | 8.98 | 0.55 | 1.03 | 0.90 | 0.71 | 0.70 | 14.68 | 5.62 | 1.41 |
| 56 | 8.54 | 0.59 | 0.91 | 1.08 | 0.97 | 0.81 | 13.62 | 4.72 | 2.25 |
| 59 | 11.23 | 0.60 | 0.90 | 0.69 | 1.04 | 1.09 | 20.79 | 4.25 | 1.65 |
| 60 | 9.35 | 0.52 | 0.78 | 0.99 | 0.87 | 1.03 | 13.41 | 5.43 | 1.21 |
| 60.1 | 10.44 | 0.54 | 0.77 | 0.82 | 1.18 | 1.01 | 18.35 | 4.46 | 1.78 |
| 60.2 | 10.54 | 0.51 | 0.85 | 1.11 | 0.91 | 0.93 | 22.90 | 6.92 | 2.31 |
| 60.3 | 10.19 | 0.55 | 0.78 | 1.09 | 0.92 | 0.91 | 22.27 | 5.57 | 2.50 |
| 61 | 13.29 | 0.49 | 0.43 | 1.25 | 0.98 | 0.80 | 20.64 | 6.19 | 1.37 |
| 62 | 11.25 | 0.57 | 0.70 | 0.97 | 1.03 | 0.83 | 21.87 | 5.13 | 2.00 |
| 63 | 10.87 | 0.49 | 0.90 | 0.84 | 1.08 | 0.85 | 22.40 | 5.17 | 2.08 |
| 63.1 | 9.25 | 0.57 | 0.95 | 0.91 | 0.94 | 0.96 | 14.97 | 4.60 | 1.79 |
| 64 | 9.33 | 0.56 | 0.72 | 1.16 | 0.90 | 0.85 | 20.04 | 3.87 | 3.00 |
| 65 | 8.24 | 0.69 | 0.81 | 1.10 | 0.89 | 0.69 | 9.82 | 8.23 | 0.95 |
| 66 | 9.73 | 0.64 | 0.42 | 1.54 | 0.92 | 0.80 | 13.10 | 6.45 | 1.41 |
| 67 | 12.57 | 0.49 | 0.84 | 0.94 | 1.19 | 0.84 | 25.64 | 7.69 | 1.93 |
| 67.1 | 15.14 | 0.56 | 0.80 | 1.04 | 0.97 | 0.86 | 32.79 | 9.08 | 2.13 |
| 67.2 | 15.88 | 0.61 | 0.78 | 1.08 | 0.87 | 0.83 | 30.14 | 10.17 | 1.69 |
| 68 | 10.25 | 0.58 | 1.11 | 0.83 | 0.96 | 0.92 | 13.53 | 5.07 | 1.45 |
| 69 | 11.64 | 0.54 | 1.07 | 0.74 | 1.03 | 1.15 | 18.56 | 5.28 | 1.52 |
| 70 | 3.71 | 0.48 | 0.81 | 0.93 | 1.11 | 1.02 | 5.01 | 3.30 | 1.13 |
|  |  |  |  |  |  |  |  |  |  |
